# *Lotus* Accessions Possess Multiple Checkpoints Triggered by Different Type III Secretion System Effectors of the Wide-Host-Range Symbiont *Bradyrhizobium elkanii* USDA61

**DOI:** 10.1264/jsme2.ME19141

**Published:** 2020-02-20

**Authors:** Shohei Kusakabe, Nahoko Higasitani, Takakazu Kaneko, Michiko Yasuda, Hiroki Miwa, Shin Okazaki, Kazuhiko Saeki, Atsushi Higashitani, Shusei Sato

**Affiliations:** 1 Graduate School of Life Sciences, Tohoku University, Miyagi, Japan; 2 Faculty of Life Sciences, Kyoto Sangyo University, Kyoto, Japan; 3 Graduate School of Agriculture, Tokyo University of Agriculture and Technology, Tokyo, Japan; 4 Department of Biological Sciences and Kyousei Science Center for Life and Nature, Nara Women’s University, Nara, Japan

**Keywords:** *Bradyrhizobium elkanii*, *Lotus japonicus*, type III secretion system, effector protein, partner selection

## Abstract

*Bradyrhizobium elkanii*, a rhizobium with a relatively wide host range, possesses a functional type III secretion system (T3SS) that is involved in symbiotic incompatibility against *Rj4*-genotype soybean (*Glycine max*) and some accessions of mung bean (*Vigna radiata*). To expand our knowledge on the T3SS-mediated partner selection mechanism in the symbiotic legume-rhizobia association, we inoculated three *Lotus* experimental accessions with wild-type and T3SS-mutant strains of *B. elkanii* USDA61. Different responses were induced by T3SS in a host genotype-dependent manner. *Lotus japonicus* Gifu inhibited infection; *L. burttii* allowed infection, but inhibited nodule maturation at the post-infection stage; and *L. burttii* and *L. japonicus* MG-20 both displayed a nodule early senescence-like response. By conducting inoculation tests with mutants of previously reported and newly identified effector protein genes of *B. elkanii* USDA61, we identified NopF as the effector protein triggering the inhibition of infection, and NopM as the effector protein triggering the nodule early senescence–like response. Consistent with these results, the *B. elkanii* USDA61 gene for NopF introduced into the *Lotus* symbiont *Mesorhizobium japonicum* induced infection inhibition in *L. japonicus* Gifu, but did not induce any response in *L. burttii* or *L. japonicus* MG-20. These results suggest that *Lotus* accessions possess at least three checkpoints to eliminate unfavorable symbionts, including the post-infection stage, by recognizing different T3SS effector proteins at each checkpoint.

Legume plants and rhizobia establish symbiosis in a unique host plant organ, the root nodule, in which rhizobia convert atmospheric dinitrogen to ammonium. Rhizobia enter the plant root hairs and develop infection threads, and rhizobia are then released from the elongated infection threads into host cells in nodule primordia. In this process, host plant roots secrete flavonoids, which activate the rhizobial transcription factor NodD. This factor induces the expression of nodulation (*nod*) rhizobial genes that are needed to produce Nod factors (NFs) (Göttfert, 1993; Perret *et al.*, 2000), which are lipochitooligosaccharides with various chemical modifications depending on the rhizobial species (Dénarié and Cullimore, 1993; Perret *et al.*, 2000). The perception of NFs by host NF receptors induces signal transduction cascades that result in nodule formation (Limpens *et al.*, 2003; Radutoiu *et al.*, 2007). Thus, interactions through the recognition of flavonoids and NFs are important for mutual recognition. In addition, rhizobial surface polysaccharides, such as lipopolysaccharides and exopolysaccharides, and their receptors in the host plant play important roles in partner selection (Becker *et al.*, 2005; Kawaharada *et al.*, 2015; Kawaharada *et al.*, 2017). Rhizobial proteins secreted by type III and IV secretion systems affect the efficiency of host plant infection (Saeki, 2011; Miwa and Okazaki, 2017).

In pathogenic bacteria, the type III secretion system (T3SS) directly injects T3SS effector proteins (T3SEs) into host cells to suppress host innate immune responses (Block *et al.*, 2008). To counteract pathogenic T3SEs, host cells have resistance (*R*) genes, such as nucleotide-binding site-leucine-rich repeat (NBS-LRR)-type genes (Gassmann and Bhattacharjee, 2012). The encoded proteins recognize T3SEs directly or indirectly, and activate immune responses called effector-triggered immunity (Gassmann and Bhattacharjee, 2012). Genome sequencing revealed that T3SS-related genes are conserved in a wide range of rhizobia including *Sinorhizobium fredii* NGR234 (Freiberg *et al.*, 1997), *S. fredii* USDA257 (Krishnan *et al.*, 2003), *S. fredii* HH103 (de Lyra Mdo *et al.*, 2006), *Mesorhizobium japonicum* MAFF303099 (reclassified from *M. loti*) (Kaneko *et al.*, 2000), *Bradyrhizobium diazoefficiens* USDA110 (Kaneko *et al.*, 2002), and *B. elkanii* USDA61 (Okazaki *et al.*, 2009). The genes encoding the rhizobial T3SS machinery components are called *rhc* (rhizobium conserved), and T3SE genes are referred to as *nop* (nodulation outer protein). The *rhc* gene cluster and the majority of *nop* genes are generally located in a symbiotic island or symbiotic plasmid (Freiberg *et al.*, 1997; Kaneko *et al.*, 2000; 2002; 2011), and the expression of these genes is controlled by NodD through the induction of the transcriptional activator TtsI (Krause *et al.*, 2002). TtsI activates the expression of *rhc* and *nop* genes through a unique *cis* element in their promoter regions called the *tts* box (Marie *et al.*, 2004; Wassem *et al.*, 2008). Rhizobial T3SEs have been reported to exert beneficial effects in the infection stage. For example, NopL of *S. fredii* NGR234 interferes with host mitogen-activated protein kinase signaling and suppresses defense reactions (Bartsev *et al.*, 2004; Zhang *et al.*, 2011). On the other hand, rhizobial T3SS is involved in incompatibility depending on the host plant genotype. In soybean (*Glycine max*), *Rj* alleles (*Rj2/Rfg1*, *Rj3*, and *Rj4*) restrict nodulation with specific rhizobial strains (Okazaki *et al.*, 2009; Hayashi *et al.*, 2012; Yasuda *et al.*, 2016; Sugawara *et al.*, 2018). The *Rj2* allele makes soybean incompatible with *B. diazoefficiens* USDA122; this incompatibility is triggered by NopP (Sugawara *et al.*, 2018). The *Rj4* allele restricts soybean nodulation with *B. elkanii* USDA61 in a T3SS-dependent manner (Okazaki *et al.*, 2009; Yasuda *et al.*, 2016). The T3SS of *B. elkanii* USDA61 is also involved in incompatibility with mung bean (*Vigna radiata*) cultivar KPS1 (Okazaki *et al.*, 2009). Despite accumulating evidence for partner selection depending on rhizobial T3SS, the underlying mechanisms remain unclear, such as the timing of effector recognition by host plants, whether T3SE conserved in several rhizobial strains affects partner selection in a single host plant, and if the same T3SE influences partner selection in different host plants.

*B. elkanii* is a microsymbiont with a relatively wide host range and induces nodules on soybean, *V. radiata*, *Arachis hypogaea* (peanut or groundnut), *V. unguiculata* (cowpea), and *Macroptilium atropurpureum* (siratro). *B. elkanii* USDA61 produces at least 10 types of NFs, including one with a similar structure to one of the NFs of the *Lotus* symbiont *M. japonicum*. (Carlson *et al.*, 1993; Niwa *et al.*, 2001). Although *B. elkanii* USDA61 cannot form nodules on *L. japonicus* Gifu accession, as reported previously (Kelly *et al.*, 2018), we demonstrated that *B. elkanii* USDA61 induced nodules on *L. japonicus* MG-20 accession. To expand our knowledge on the T3SS-mediated partner selection mechanism, we herein focused on the model legume *L. japonicus* and a related species, *L. burttii*, and inoculated them with wild-type *B. elkanii* USDA61 and its T3SS-deficient mutant.

## Materials and Methods

### Bacterial strains

The bacterial strains and plasmids used in the present study are listed in Tables 1 and 2. *B. elkanii* strains and *M. japonicum* strains were cultured at 28°C in arabinose–gluconate (AG) medium (Sadowsky *et al.*, 1987) or tryptone-yeast extract medium (Beringer, 1974) supplemented with appropriate antibiotics (Table 1). *Escherichia coli* strains were cultured at 37°C in Luria–Bertani medium (Sambrook and Russell, 2001) supplemented with appropriate antibiotics (Table 1).

### Plant materials, growth conditions, and inoculation

*L. japonicus* experimental accessions Gifu (B-129) and MG-20, *L. burttii*, and the *nfr1* mutant (Gifu background) were used (Hashiguchi *et al.*, 2018). *Lotus* seeds were scarified with sandpaper, sterilized with solution containing 2% (v/v) sodium hypochlorite and 0.02% (v/v) Tween-20 for 10‍ ‍min, rinsed five times with sterilized distilled water, and germinated on 0.8% (w/v) agar plates for 3‍ ‍d in the dark, followed by 1‍ ‍d under light with a 16-h day and 8-h night condition. Seedlings were transferred to autoclaved vermiculite in inoculation pots (7 to 16 plants pot^–1^) with nitrogen-free B&D medium (Broughton and Dilworth, 1971).

Rhizobial cultures were incubated for 3 to 5‍ ‍d, centrifuged (8,000×*g*, room temperature, 2‍ ‍min), washed three times with sterilized distilled water, suspended in nitrogen-free B&D medium, and 20 mL of the inoculant (OD_600_=0.1) was then added to each pot containing seedlings. Plants were grown in a growth chamber at 25°C with a 16-h day and 8-h night condition. Nodule numbers and nodule fresh weights were measured on day 30 post-inoculation.

### Analysis of proteins secreted by T3SS

AG medium (Sadowsky *et al.*, 1987) (120 mL) inoculated with a 1:100 dilution of the *B. elkanii* preculture was incubated at 28°C for 48 h in the presence of 10‍ ‍μM genistein, which activates the expression of the T3SS machinery and T3SE genes in *Bradyrhizobium* species (Hempel *et al.*, 2009). Extracellular proteins were collected from the culture supernatant as follows. The bacterial culture was centrifuged twice at 4°C (4,000×*g* for 1 h; 8,000×*g* for 30‍ ‍min) to remove cells and exopolysaccharides, and 100 mL of the culture supernatant was collected. Aliquots of the supernatant (25 mL each) were dispensed into two 50-mL centrifuge tubes, and 7.5 mL of Tris-EDTA-saturated phenol and 1 mL of 1 M dithiothreitol were then added. The mixture was vortexed and centrifuged (10,000×*g*, room temperature, 30‍ ‍min). The water phase was removed, and the remaining culture supernatant (25 mL each) and 1 mL of 1 M dithiothreitol were added to the phenol phase. The mixture was vortexed and centrifuged again (10,000×*g*, room temperature, 30‍ ‍min). The phenol phase was collected and mixed with 20 mL methanol, 300‍ ‍μL 8 M ammonium acetate, and 400‍ ‍μL 1 M dithiothreitol. The mixture was incubated at –20°C overnight. Proteins were pelleted by centrifugation (10,000×*g*, 4°C, 1 h), washed with 70% ethanol, combined from both tubes into a 5-mL Eppendorf tube, and suspended in 50‍ ‍μL phosphate-buffered saline. Protein concentrations were measured using a Bradford-based method. Proteins (5‍ ‍μg) were then separated by SDS-PAGE in precast 5–20% gradient gels (HOG-0520; Oriental Instruments) and stained with Coomassie Brilliant Blue R250. T3SS-dependent bands were subjected to in-gel digestion with trypsin. A thin matrix layer was made with 1‍ ‍μL of α-cyano-4-hydroxy-cinnamic acid (CHCA) solution (1 mg mL^–1^ CHCA in 50% acetonitrile containing 0.1% TFA and 25‍ ‍mM ammonium bicarbonate) on a sample plate (Sciex). Aliquots (1‍ ‍μL) of tryptic peptides were dropped onto the thin layer, air dried, and covered with 1‍ ‍μL of CHCA solution. Mass spectrometry was performed on a TOF/TOF 5800 mass spectrometer (Sciex). Database searches for protein identification were performed using MS-Fit (http://prospector.ucsf.edu) and the BE61 protein database (Kaneko *et al.*, unpublished).

In a large-scale analysis of extracellular proteins with the iTRAQ system, *B. elkanii* USDA61 and the *rhcJ* gene disruptant (BErhcJ) were cultured in the presence of 10‍ ‍μM genistein. Extracellular proteins (20‍ ‍μg) from each culture supernatant were labeled using an iTRAQ Reagent Multi-Plex Kit (Sciex). Proteins were digested with trypsin and the labeled peptides were loaded onto a cation exchange spin column (Viva S Mini H; Sartorius) and eluted with 150‍ ‍mM or 1 M KCl in 10‍ ‍mM potassium phosphate and 20% (v/v) acetonitrile at pH 3.0. Acetonitrile was evaporated and aliquots were loaded onto a C18 tip column (Rappsilber *et al.*, 2007) for desalting and stored on the column at –80°C until used. Peptides were separated on a C18W-3 column (DiNa nano-LC system; KYA Technologies). A mass spectrometric analysis was performed using TOF/TOF 5800. The database search and relative quantitation were performed using ProteinPilot (Sciex) and the BE61 protein database.

### Construction of bacterial strains

DsRed-labeled rhizobial strains were constructed using a previously described method (Hayashi *et al.*, 2014) and the DsRed transposon delivery vector pBjGroEL4::DsRed2, in which the DsRed-coding sequence was fused to the promoter region of the *groEL4* gene from *B. japonicum* USDA110, and *groEL4* promoter-driven DsRed was integrated into the mini transposon mini-Tn5.

*B. elkanii nopL*, *nopP1*, *nopP2*, and *nopM* mutants were constructed by single crossover recombination as described previously (Faruque *et al.*, 2015) with the primers listed in Table 2. *B. elkanii Be61_78180* and *nopF* mutants were constructed as follows. The internal regions of *Be61_78180* and *BenopF* were amplified by a polymerase chain reaction (PCR) with the primer pairs BE61_78180int-NotI-L and BE61_78180int-SpeI-R, and nopFint-NotI-L and nopFint-SpeI-R, respectively (Table 2). PCR products were digested with the restriction enzymes NotI and SpeI and cloned into the NotI and SpeI sites of the pSUPSCAKm vector (Okazaki *et al.*, 2009). The plasmids obtained (pSUPSCAKm-*Be61_78180* and pSUPSCAKm-*nopF*, respectively) were introduced into *E. coli* DH5α (Toyobo). To transfer the two plasmids into *B. elkanii* USDA61, we used a bacterial conjugation system as follows. Each bacterial culture (1 mL) was centrifuged (8,000×*g*, room temperature, 2‍ ‍min), and the pellet was washed twice with sterilized distilled water and suspended in 1 ml of AG medium. One hundred microliters of the donor strain (*E. coli* harboring pSUPSCAKm-*Be61_78180* or pSUPSCAKm-*nopF*), 100‍ ‍μL of the helper strain (*E. coli* harboring pRK2013), and 300‍ ‍μL of the recipient strain (*B. elkanii* USDA61) were mixed and centrifuged (8,000×*g*, room temperature, 2‍ ‍min). Each pellet was suspended in 60‍ ‍μL AG medium, dropped onto an AG plate, and incubated at 28°C for 2 d. Cells were collected and single-crossover mutants were selected on an AG plate containing 50‍ ‍μg mL^–1^ polymyxin and 200‍ ‍μg mL^–1^ kanamycin. The integration of pSUPSCAKm-*BE61_78180* and pSUPSCAKm-*nopF* into the internal regions of *BE61_78180* and *BenopF*, respectively, was confirmed by PCR.

The *BenopM* and *BenopF* genes were introduced separately into *M. japonicum* MAFF303099 or the T3SS-disrupted *M. japonicum* strain DT3S (Okazaki *et al.*, 2010) as follows. The 2,939-bp fragment containing the coding region and *tts* box promoter region of *BenopM* and the 666-bp fragment containing those of *BenopF* were amplified by PCR using the primer pairs nopM-NotI-L and nopM-NotI-R, and nopF-NotI-L and nopF-NotI-R, respectively (Table 2). PCR products were digested with the restriction enzyme NotI and cloned into the NotI site of the GFP-expressing plasmid pHC60 (Cheng and Walker, 1998). The plasmids obtained (pHC60-*BenopM* and pHC60-*BenopF*, respectively) were introduced separately into *E. coli* DH5α and mobilized into *M. japonicum* MAFF303099 using the bacterial conjugation system described above. One-day post conjugation, transformants containing the *BenopM* or *BenopF* gene were selected on tryptone-yeast extract plates containing 100‍ ‍μg mL^–1^ phosphomycin and 2.0‍ ‍μg mL^–1^ tetracycline. The transfer of pHC60-*BenopM* or pHC60-*BenopF* was confirmed by PCR.

Regarding *BenopM* complementation, *BenopM* and its upstream *tts* box region were amplified by PCR with the primers nopM-SacI-R and nopM-SacI-L, and cloned into the mini-Tn5 region of the pBjGroEL4::DsRed2 plasmid. The resulting plasmid, Tn5::nopM, was mobilized into BEnopM using the bacterial conjugation system. The integration of mini-Tn5 containing *BenopM* into the USDA61 genome was confirmed by antibiotic resistance and PCR.

Regarding *BenopF* complementation, the *BenopF* gene, its promoter region, and a 1,281-bp non-coding region of the USDA61 genome were amplified by PCR using the primer sets pS18mob_EcoR1_inf, nopF_inf_1 and nopF_inf_2, and nopF_inf_3, and cloned into the *Eco*RI sites of the pS18mob plasmid (Okazaki *et al.*, unpublished) using an In-fusion HD Cloning Kit (Takara Bio). The resulting plasmid, pS18mob-nopF, was mobilized into BEnopF using the bacterial conjugation system. The single-crossover recombination of pS18mob-nopF in the non-coding region was confirmed by antibiotic resistance and PCR.

### Microscopy

Root nodules were observed under a stereomicroscope (SZ61; Olympus), and DsRed-fluorescent nodules, nodule sections, and infection threads under a fluorescence microscope (SMZ18; Nikon). Early infection events were observed under a confocal microscope (LSM800; Zeiss).

### Data availability

Nucleotide sequences have been submitted to the DNA Data Bank of Japan (DDBJ) with the accession numbers LC471584 (*Be61_78180*), LC471585 (*BenopM*), and LC471586 (*BenopF*).

## Results

### T3SS of *B. elkanii* USDA61 induces three types of responses in *Lotus* accessions

**Fig. 1.**
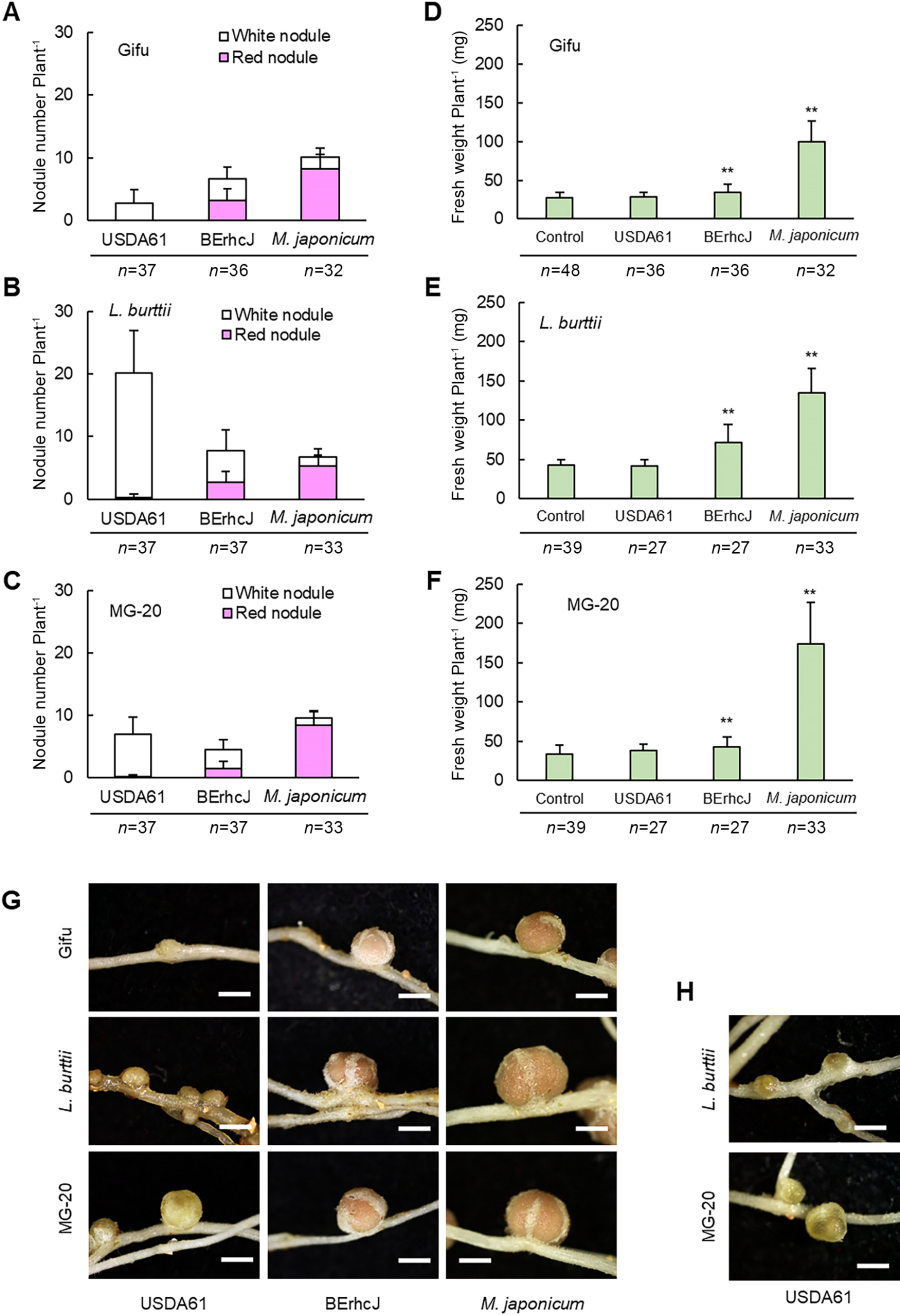
Phenotypes of *Lotus* accessions inoculated with *B. elkanii* USDA61, the T3SS-deficient BErhcJ mutant of *B. elkanii*, or *M. japonicum* MAFF303099. (A, B, and C) Nodule numbers and (D, E, and F) fresh weights of *Lotus japonicus* Gifu (A, D), *L. burttii* (B, E), and *L. japonicus* MG-20 (C, F) inoculated with the wild-type (USDA61) or T3SS-deficient mutant (BErhcJ) of *B. elkanii* USDA61, or *Lotus* symbiont *M. japonicum* MAFF303099 measured on day 30 post-inoculation. Measurements were performed three times with 6 to 16 plants each time. In panels B and C, brownish nodules were included in the count of white nodules. Error bars indicate standard deviations. The Student’s *t*-test was performed for fresh weight comparisons; ** *P*<0.01 vs. control (no inoculation). (G) Root nodules of the three *Lotus* accessions inoculated with the above bacteria. Scale bars=1 mm. (H) Brownish nodules of *L. burttii* and MG-20 inoculated with wild-type USDA61. Scale bars=1 mm.

To investigate the symbiotic potential of *B. elkanii* USDA61 for *Lotus*, we inoculated *L. japonicus* Gifu (B-129), *L. japonicus* MG-20 (Miyakojima), and *L. burttii* with wild-type USDA61. Only a few white nodules formed on Gifu, a few well-developed red nodules (mature nodules) formed on MG-20, and many small white nodules and few red nodules formed on *L. burttii* (Fig. 1A, B, C and G). Some of the developed and small nodules on *L. japonicus* MG-20 and *L. burttii* were brownish (Fig. 1H), resembling the phenotype of nodule early senescence (Yamaya-Ito *et al.*, 2018). To test the effects of T3SS of *B. elkanii* USDA61, we inoculated *Lotus* accessions with BErhcJ, a strain carrying a mutation in the *rhcJ* gene encoding a T3SS machinery component (Okazaki *et al.*, 2009). Mature nodules formed on all three *Lotus* accessions, indicating that phenotypic differences were caused by the T3SS of *B. elkanii*; however, nodule numbers and plant growth (fresh weights) were less than those induced by the inoculation with the *Lotus* symbiont *M. japonicum* MAFF303099 (Fig. 1D, E, F and S1). To investigate whether NFs are needed for nodulation by USDA61, we inoculated wild-type Gifu and the nod factor receptor 1 mutant (*nfr1*) with USDA61 or BErhcJ; the latter induced the formation of mature nodules in wild-type Gifu, but not in *nfr1*, indicating that nodulation by USDA61 depends on nod factor recognition (Fig. S2).

**Fig. 2.**
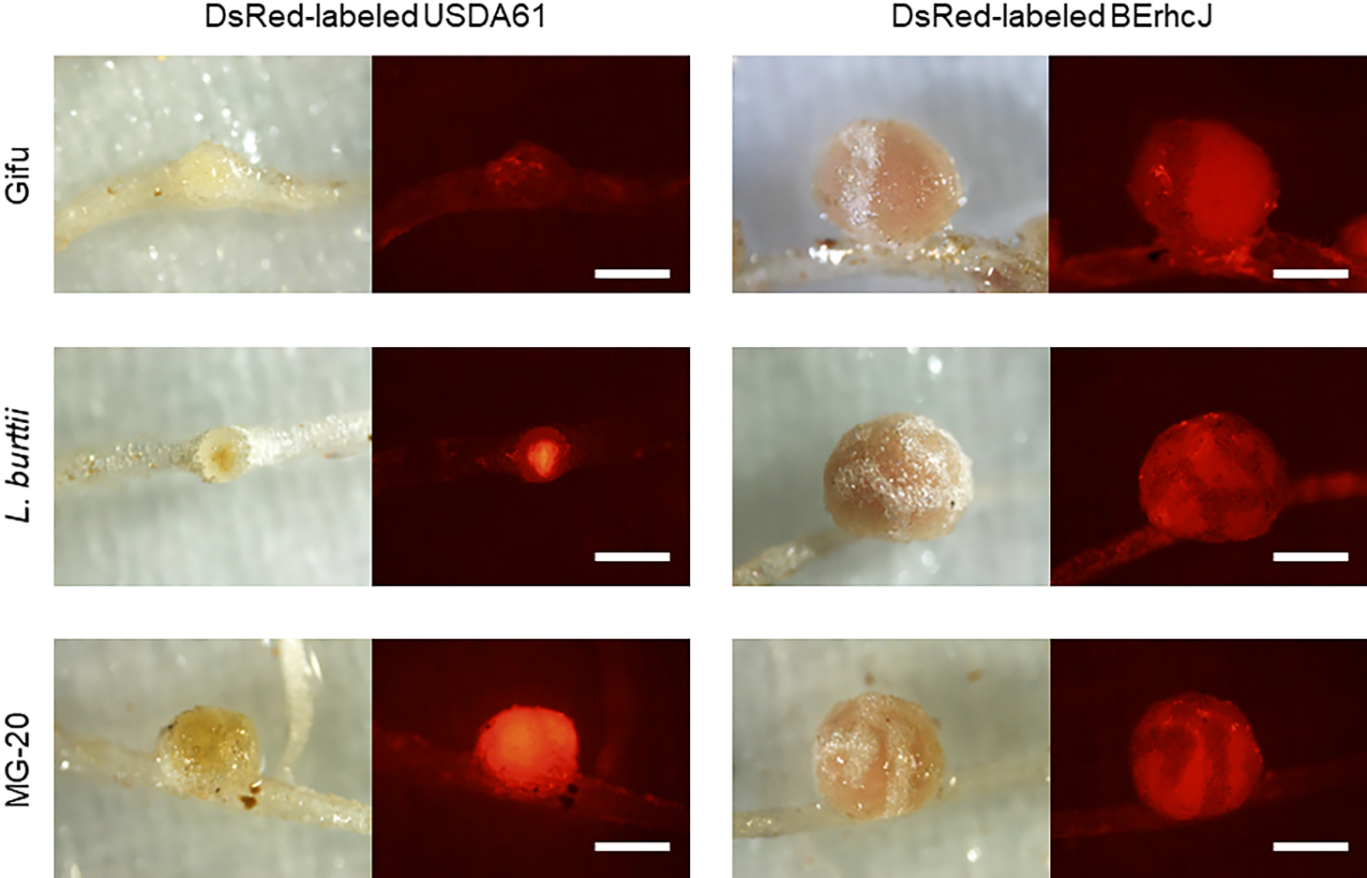
Infection phenotypes of *Lotus* accessions inoculated with *B. elkanii* USDA61 or the T3SS-deficient mutant BErhcJ. *L. japonicus* Gifu, *L. burttii*, and *L. japonicus* MG-20 were inoculated with two DsRed-labeled rhizobial strains, and DsRed fluorescence in root nodules was observed under a fluorescence microscope on day 30 post-inoculation. Scale bars=1 mm.

The inoculation with DsRed-labeled USDA61 led to fluorescence of the entire nodules of *L. japonicus* MG-20 and a limited area of *L. burttii* nodules, but no clear fluorescence in *L. japonicus* Gifu (Fig. 2), as was reported previously for an inoculation of *Rj4*-genotype soybean in which infection inhibition was induced (Yasuda *et al.*, 2016). The inoculation with DsRed-labeled BErhcJ led to fluorescence of the entire nodules in all three accessions (Fig. 2). By sectioning the nodules, it was confirmed that fluorescence observed in *L. japonicus* MG-20 and *L. burttii* inoculated with DsRed-labeled USDA61 as well as DsRed-labeled BErhcJ came from inside the nodules (Fig. S3). These results, together with those shown in Fig. 1, suggest that the T3SEs of USDA61 influence(s) responses at the post-infection stage, *i.e.*, nodule maturation inhibition (rhizobia may infect, but nodules remain small and white even after day 30 post-inoculation) in *L. burttii* and a nodule early senescence–like response in *L. burttii* and *L. japonicus* MG-20.

### NopM induces a nodule early senescence–like response

**Fig. 3.**
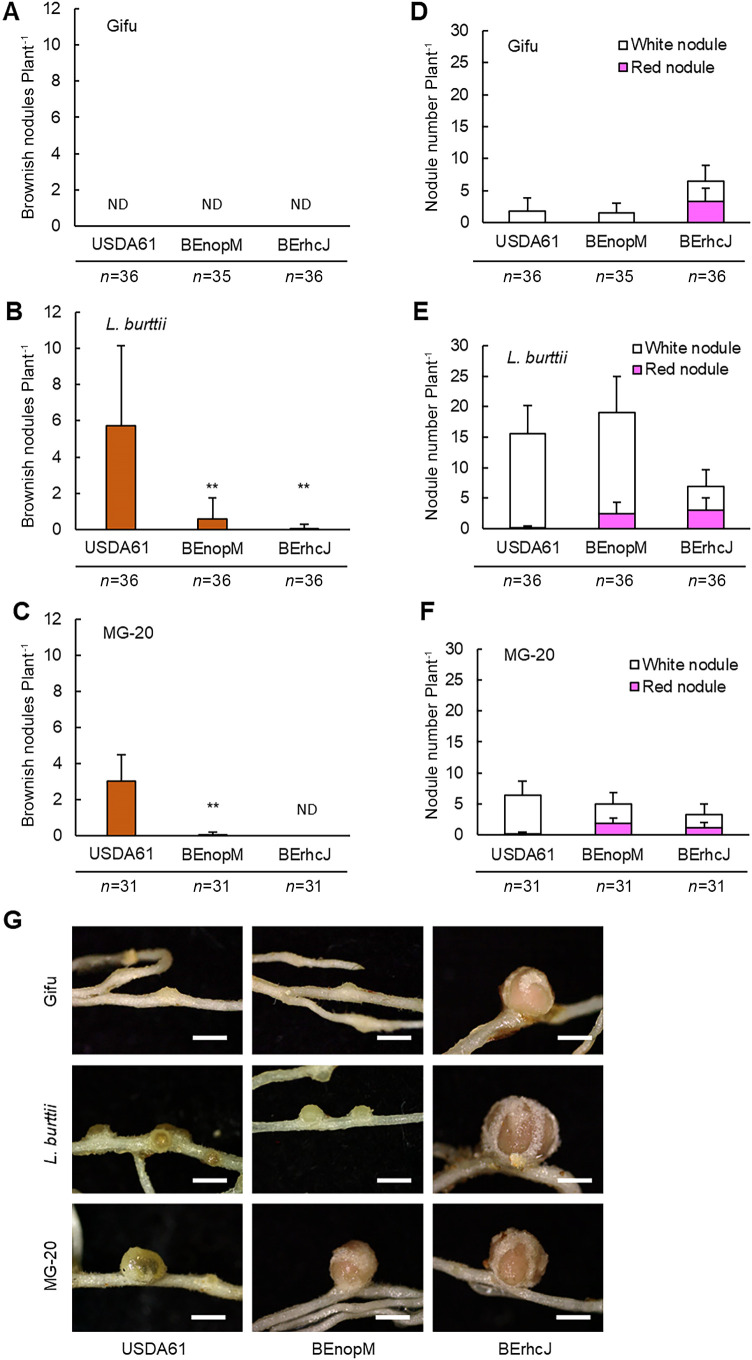
Characterization of the NopM protein of *B. elkanii* USDA61. Phenotypes of three *Lotus* accessions inoculated with wild-type *B. elkanii* USDA61, the *BenopM* mutant (BEnopM), or T3SS-deficient mutant (BErhcJ) were analyzed on day 30 post-inoculation. (A, B, and C) The number of brownish nodules and (D, E, and F) total number of mature and white nodules on the roots of (A, D) Gifu, (B, E) *L. burttii*, and (C, F) MG-20 are shown. ND means not detected. Inoculation tests were performed three times with 7 to 12 plants each time. In panels E and F, brownish nodules were included in the count of white nodules. Error bars indicate standard deviations. The Student’s *t*-test was performed for brownish nodule counts; ** *P*<0.01 vs. wild-type USDA61. (G) Root nodules of the three *Lotus* accessions inoculated with the above bacteria. Scale bars=1 mm.

To identify the T3SEs involved in these checkpoint responses of the *Lotus* accessions, we investigated the interaction of the reported T3SEs of USDA61 with the *Lotus* accessions. Since NopP, NopL, and NopM were confirmed as T3SEs in USDA61 (Okazaki *et al.*, 2009) at the time of the experiment, we constructed mutants of the two copies of *BenopP*, BEnopP1 and BEnopP2; a *BenopL* mutant, BEnopL; and a *BenopM* mutant, BEnopM. The inoculation with BEnopP1, BEnopP2, or BEnopL resulted in similar nodulation phenotypes of the three *Lotus* accessions to those induced by wild-type USDA61 (Fig. S4). However, *L. burttii* and MG-20 had fewer brownish nodules after the inoculation with BEnopM than with wild-type USDA61 (Fig. 3B, C, E, F, and G) or BEnopM complemented with the *BenopM* gene (Fig. S5). The inoculation with BEnopM slightly improved plant growth by *L. burttii* and *L. japonicus* MG-20 (Fig. S6). The inoculation with BEnopM did not change the nodulation phenotype of *L. japonicus* Gifu and did not alter the number of white nodules on *L. burttii* from those with the inoculation with wild-type USDA61 (Fig. 3A, D, E, and G). These results suggest that NopM induces a nodule early senescence-like response in *L. burttii* and *L. japonicus* MG-20, and that infection inhibition in *L. japonicus* Gifu and maturation inhibition in *L. burttii* are induced by T3SEs other than NopP, NopL, and NopM. The product of *BenopM* predicted from the genome sequence is composed of 610 amino acids. The domain organization and phylogenetic relationships of NopM are summarized in Fig. S7.

### Field-isolated *B. elkanii* strain lacking two effector proteins evades infection inhibition by *L. japonicus* Gifu

**Fig. 4.**
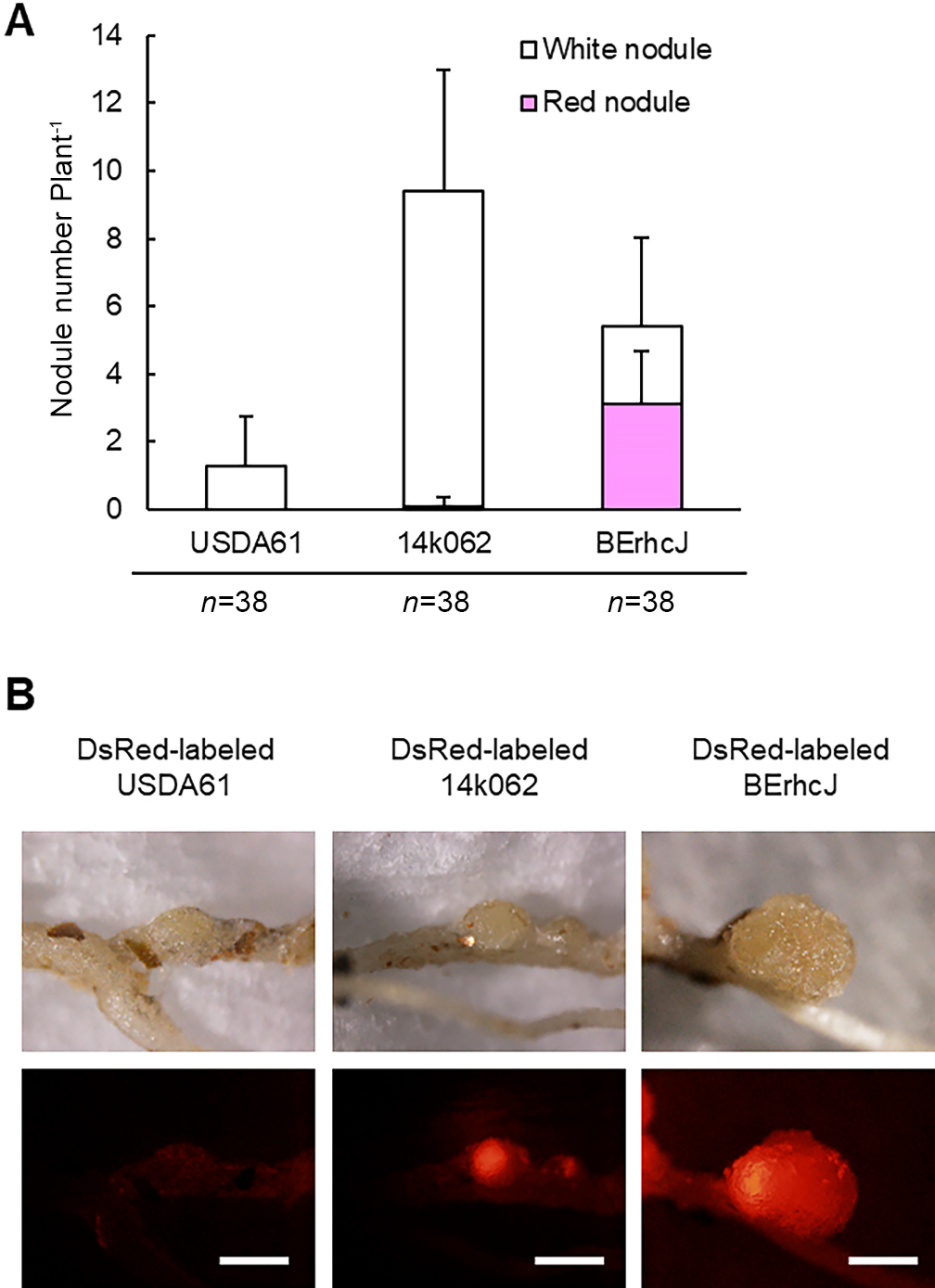
Symbiotic phenotype of *B. elkanii* 14k062. (A) Nodule number of *L. japonicus* Gifu inoculated with wild-type *B. elkanii* USDA61, 14k062 (a field-isolated strain of *B. elkanii*), or the T3SS-deficient mutant (BErhcJ) on day 30 post-inoculation. Nodulation tests were performed three times with 12 to 14 plants each time. Error bars indicate standard deviations. (B) Infection phenotypes of *L. japonicus* Gifu inoculated with DsRed-labeled USDA61, 14k062, or BErhcJ on day 30 post-inoculation. DsRed fluorescence was observed under a fluorescence microscope. Scale bars=1 mm.

During the course of the large-scale field phenotyping of *Lotus* accessions (Shah *et al.*, 2020), we isolated *B. elkanii* strains, confirmed by 16S rDNA sequences, from the nodules of *L. japonicus* accessions grown in the Kashimadai field (Osaki city, Miyagi, Japan) on which soybean had been cultivated over a three-year period. Strain 14k062 isolated from *L. japonicus* Gifu induced many white nodules on Gifu roots (Fig. 4A), resembling the phenotype of *L. burttii* inoculated with USDA61 (Fig. 1). The inoculation of Gifu with the DsRed-labeled 14k062 strain resulted in clear, but limited, areas of DsRed fluorescence within the nodules (Fig. 4B), similar to those in nodules on *L. burttii* inoculated with USDA61 (Fig. 2). On the other hand, the inoculation of *L. burttii* and *L. japonicus* MG-20 with 14k062 caused almost the same nodulation phenotypes as the inoculation with USDA61 (data not shown). These results suggest that 14k062 has the T3SS machinery, but lacks the T3SE(s) that trigger infection inhibition.

**Fig. 5.**
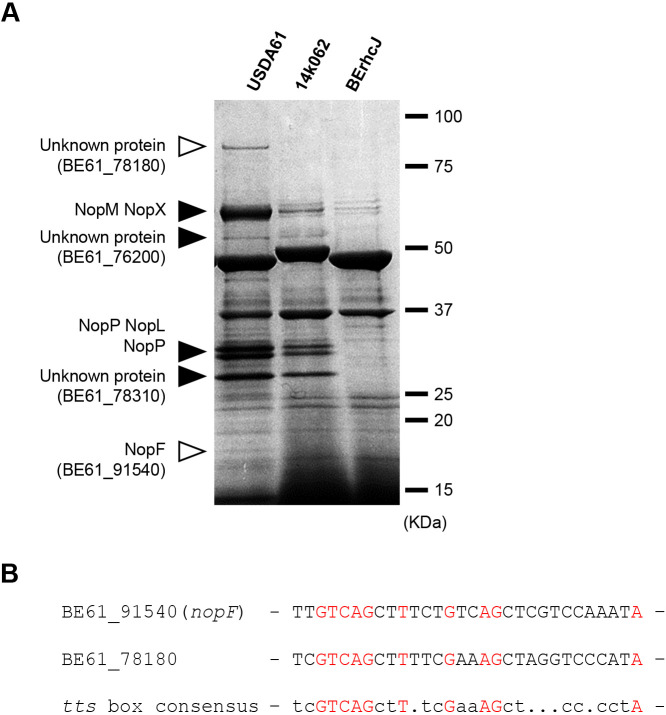
Proteins secreted by *B. elkanii* strains. *B. elkanii* strains USDA61 and 14k062 and the T3SS-deficient mutant (BErhcJ) were grown in the presence of 10‍ ‍μM genistein. Supernatants containing extracellular proteins were collected, and proteins were separated by SDS-PAGE (5 to 20% gradient gel) and stained with Coomassie Brilliant Blue. Closed arrowheads indicate T3SS-dependent secreted proteins, and open arrowheads indicate proteins not detected in the 14k062 strain. (B) The *tts* box sequences of *nopF* and *Be61_78180* of *B. elkanii* USDA61. In the consensus sequence, all invariant nucleotides are capitalized and lowercase letters are used for nucleotides conserved in at least 50% of the analyzed sequences (Krause *et al.*, 2002). Nucleotides in common with the consensus sequence are shown in red.

To analyze T3SEs lacking in the 14k062 strain, we attempted to create a comprehensive list of T3SEs in USDA61 by comparing the extracellular proteins of wild-type USDA61 and BErhcJ using a MALDI-TOF-MS/MS analysis with the iTRAQ protein labeling system (Ross *et al.*, 2004). We identified 9 candidate T3SEs based on their presence in wild-type USDA61 and low abundance or absence in BErhcJ (BErhcJ/wild-type USDA61 ratio <0.2) (Table 3 and matched peptides are shown in Table S1). These proteins included previously identified effector proteins (NopL, two isoforms of NopP, and NopM) and T3SS machinery components (NopA and NopX) (Okazaki *et al.*, 2009). The new candidates were BE61_51850, BE61_76200, BE61_78180, BE61_78310, and BE61_91540 (Table 3). We then compared extracellular proteins between USDA61 and 14k062 by separating them electrophoretically, and confirmed the presence of most of the T3SE candidates in the 14k062 culture supernatant, indicating that 14k062 is not a mutant of a T3SS machinery component as expected (Fig. 5A). Two T3SE candidates were not detected in the 14k062 culture supernatant (Fig. 5A); using the MALDI-TOF-MS/MS analysis, we identified them as BE61_78180 and BE61_91540 (Table 3). The promoters of the corresponding two genes contained a typical *tts* box (Fig. 5B). A comparison of the amino acid sequence of BE61_91540 with the rhizobium genome data set in RhizoBase (http://genome.annotation.jp/rhizobase) revealed that BE61_91540 is conserved among *B. diazoefficiens* USDA110, *B. diazoefficiens* USDA122, and *B. japonicum* USDA6, and is annotated as T3SS-secreted protein NopF with no functional information (Hempel *et al.*, 2009; Kimbrel *et al.*, 2013; Tsukui *et al.*, 2013). Based on these findings, we considered infection inhibition in *L. japonicus* Gifu to be triggered by BE61_78180 and/or NopF (BE61_91540).

### NopF triggers infection inhibition in *L. japonicus* Gifu

**Fig. 6.**
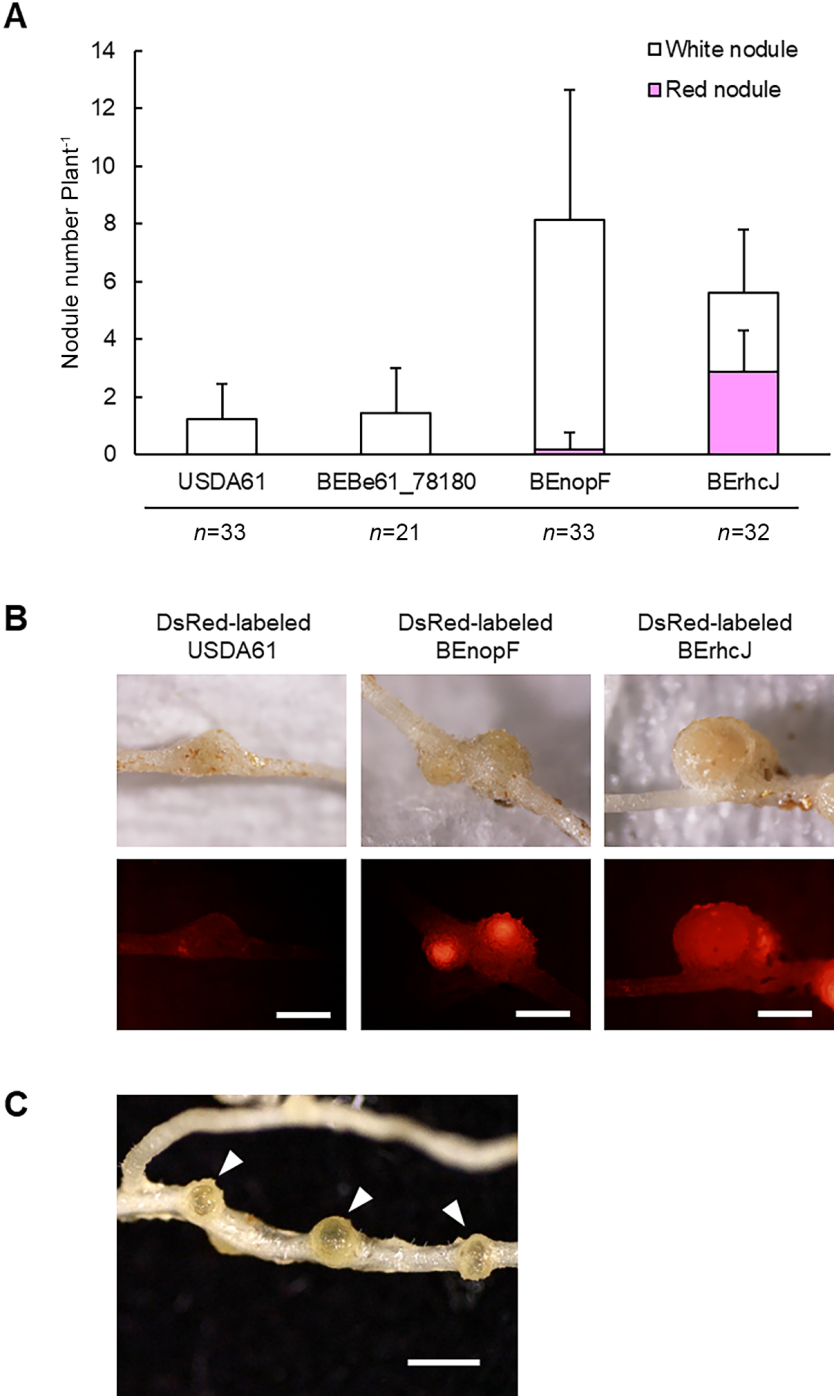
Characterization of NopF of *B. elkanii* USDA61. (A) Nodule numbers of *L. japonicus* Gifu inoculated with wild-type *B. elkanii* USDA61, *Be61_78180* mutant (BEbe61_78180), *BenopF* mutant (BEnopF), or T3SS-deficient mutant (BErhcJ) on day 30 post-inoculation. Nodulation tests were performed at least twice with 9 to 12 plants each time. Error bars indicate standard deviations. (B) Infection phenotype of *L. japonicus* Gifu inoculated with DsRed-labeled BEnopF strain on day 30 post-inoculation. DsRed fluorescence was observed under a fluorescence microscope. Scale bars=1 mm. (C) Brownish nodules on *L. japonicus* Gifu inoculated with BEnopF. Scale bar=1 mm.

To identify which of these two proteins triggers infection inhibition in *L. japonicus* Gifu, we constructed mutants of the *Be61_78180* and *BenopF* genes, and used them to inoculate *L. japonicus* Gifu. The phenotype induced by the *Be61_78180* mutant (BEbe61_78180) did not significantly differ from that induced by the USDA61 inoculation (Fig. 6A), whereas the *BenopF* mutant (BEnopF) induced many small white nodules (Fig. 6A), at a similar level to that induced by 14k062 (Fig. 4A). The inoculation of Gifu with DsRed-labeled BEnopF clearly showed the release of BEnopF into the nodules (Fig. 6B), whereas the nodules were rarely mature and most of them became brownish (Fig. 6C), as was the case in *L. burttii* inoculated with wild-type USDA61. These results suggest that *L. japonicus* Gifu inhibits nodule maturation and has an early senescence-like response. Consistent with this observation, the growth of *L. japonicus* Gifu inoculated with BEnopF was similar to that after the inoculation with wild-type USDA61 (Fig. S8). The inoculation of *L. burttii* with BEnopF, BEbe61_78180, or wild-type USDA61 resulted in a similar phenotype (Fig. S9), and *L. burttii* and MG-20 inoculated with BEnopF or BEbe61_78180 also showed a nodule early senescence-like response (Fig. S10). These results indicate that NopF, not BE61_78180 triggers infection inhibition in *L. japonicus* Gifu, and neither of these proteins triggers nodule maturation inhibition or an early senescence-like response. The complementation test on the *BenopF* gene confirmed that infection inhibition was induced by NopF (Fig. S11).

Nodulation restrictions against rhizobial T3SS are generally induced during infection thread formation (Yasuda *et al.*, 2016). To investigate whether this is the case for infection inhibition induced by NopF, we inoculated Gifu with DsRed-labeled USDA61, BEnopF, BErhcJ, or *M. japonicum* MAFF303099, and counted infection threads on day 10 post-inoculation. Well-elongated infection threads were observed after the *M. japonicum* inoculation, whereas no infection threads were detected after the inoculation with *B. elkanii* strains, including BEnopF and BErhcJ (Fig. S12A, B, and C). Confocal observations of the nodule on day 14 post-inoculation with *M. japonicum* showed well-elongated infection threads on nodules, whereas the attachment of BEnopF and BErhcJ bacteria to the nodule surface and entry toward the nodule center were noted with no obvious infection threads (Fig. S12D). After the wild-type USDA61 inoculation, only the attachment of bacteria to the nodule surface was observed (Fig. S12D). This result suggests that *B. elkanii* infects Gifu by crack entry rather than through infection threads, and that NopF triggers infection inhibition in this process.

The predicted product of *BenopF* has 179 amino acids and belongs to the Acyl-CoA N-acyltransferase superfamily (InterPro ID; IPR016181). A BLASTP analysis showed that the amino acid sequence of NopF was identical to those of proteins encoded by the two gene copies in *B. diazoefficiens* USDA110 (Bll1862 and Bll8201) and USDA122 (BD122_09540 and BD122_41920), and by a single-copy gene in *B. japonicum* USDA6 (BJ6T_88790). A homolog of the *nopF* gene was not conserved in *M. japonicum* MAFF303099, similar to the *nopM* gene. NopF was 44% identical to the HopBG1 protein, a T3SE of the plant pathogen *Pseudomonas syringae* pv. *maculicola* ES4326 (Baltrus *et al.*, 2011). The features of NopF are summarized in Fig. S13.

### NopF secreted by *M. japonicum* MAFF303099 induces infection inhibition in *L. japonicus* Gifu

**Fig. 7.**
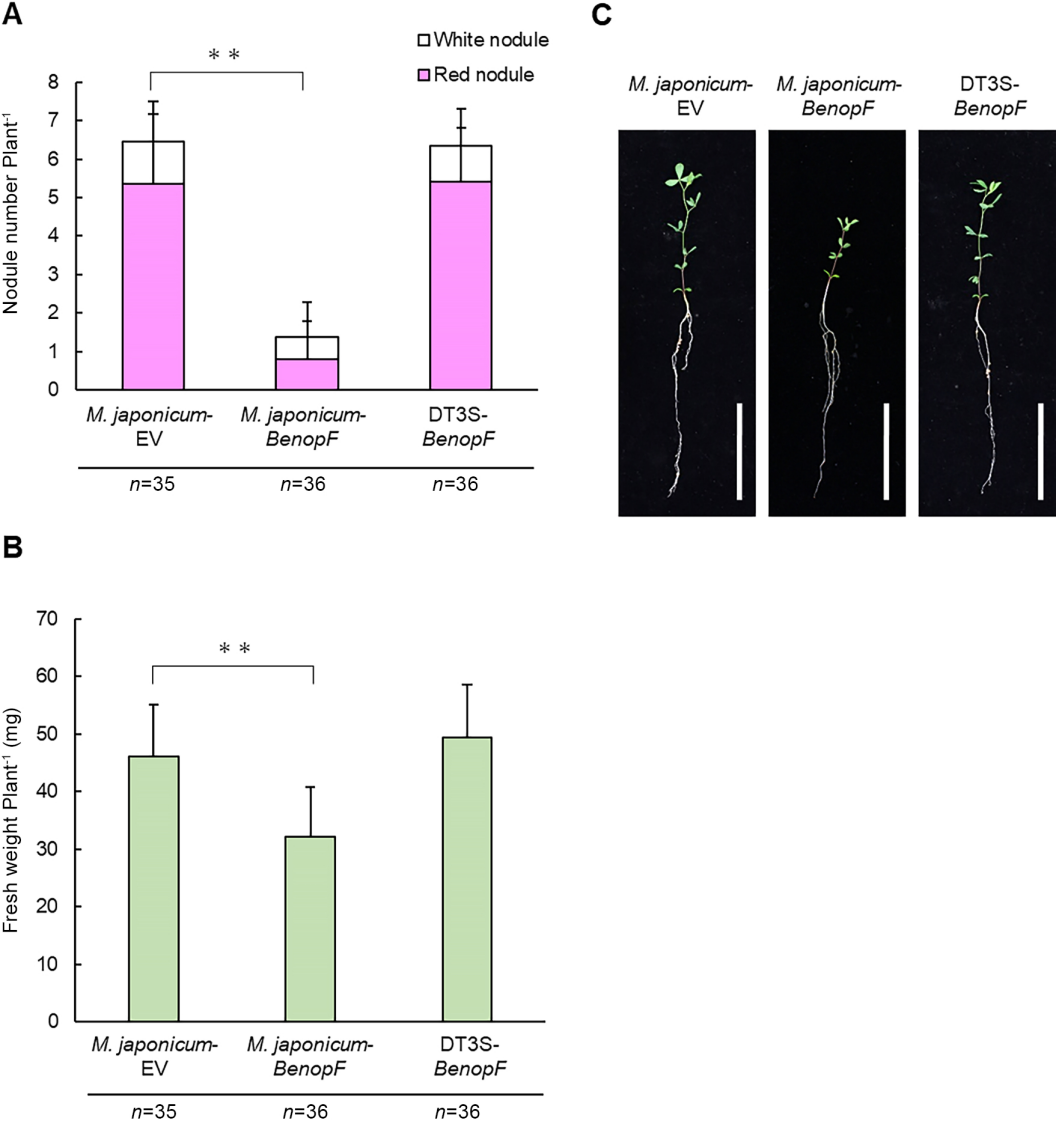
Symbiotic phenotypes of *L. japonicus* Gifu inoculated with *M. japonicum* MAFF303099 carrying NopF of *B. elkanii* USDA61. (A) Nodule numbers and (B) nodule fresh weights of plants inoculated with *M. japonicum* carrying the plasmid pHC60 (*M. japonicum*-EV), *M. japonicum* carrying pHC60-*BenopF* (*M. japonicum*-*BenopF*), or the *M. japonicum* T3SS-deficient mutant carrying pHC60-*BenopF* (DT3S-*BenopF*) on day 21 post-inoculation. All tests were performed three times with 11 to 12 plants each time. Error bars indicate standard deviations. ** *P*<0.01 vs. *M. japonicum*-EV in the Student’s *t*-test. (C) Plant growth phenotype of *L. japonicus* Gifu inoculated with the above bacteria. Scale bars=5 cm.

To elucidate the functions of NopM and NopF effector proteins, we introduced *BenopM* and *BenopF* cloned into the GFP constitutively-expressing plasmid pHC60 (Cheng and Walker, 1998) into *M. japonicum* MAFF303099 to generate *M. japonicum-BenopM* and *M. japonicum-BenopF*, respectively. We expected the two proteins to be functional because *M. japonicum* has the T3SS machinery (Kaneko *et al.*, 2000; Okazaki *et al.*, 2010), and *B. elkanii* USDA61 and *M. japonicum* MAFF303099 share a similar typical *tts* box promoter (Fig. S14). As a control, we used *M. japonicum* carrying empty pHC60 (*M. japonicum*-EV). On day 21 post-inoculation, the total nodule number and plant fresh weight of *L. japonicus* Gifu were markedly lower in plants inoculated with *M. japonicum-BenopF* than with *M. japonicum*-EV (Fig. 7A, B, and C). Microscopic observations revealed that the number of infection threads were lower with the *M. japonicum-BenopF* inoculation than with the *M. japonicum*-EV inoculation (Fig. S15). To confirm the T3SS dependence of NopF secretion by *M. japonicum*, we introduced the *BenopF* plasmid into the T3SS-disrupted *M. japonicum* strain, DT3S (Okazaki *et al.*, 2010). Plants inoculated with DT3S-*BenopF* formed mature nodules and grew similarly to those inoculated with *M. japonicum-*EV (Fig. 7A, B, and C). These results indicate that introduced NopF was secreted into the host plant through *M. japonicum* T3SS, and its secretion triggered infection inhibition in *L. japonicus* Gifu. In *L. burttii* and *L. japonicus* MG-20, *M. japonicum-BenopF* induced mature nodules at the same level as *M. japonicum*-EV, and plant fresh weight did not significantly differ between the *M. japonicum-BenopF* and *M. japonicum*-EV inoculations, as expected from the phenotypes of wild-type USDA61 and the BEnopF inoculation (Fig. S16).

In contrast, mature nodules, but not brownish nodules, were induced by *M. japonicum-BenopM* in all three *Lotus* accessions (Fig. S17). This result suggests that the introduction of NopM did not occur in *M. japonicum* or that introduced NopM did not function in *L. japonicus* cells.

## Discussion

**Fig. 8.**
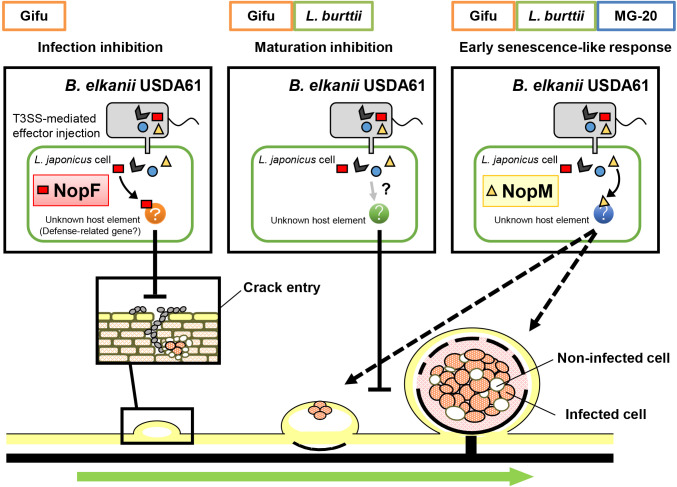
Model of the T3SS-mediated interaction between *B. elkanii* USDA61 and three *Lotus* accessions. Three types of *Lotus* responses—infection inhibition, nodule maturation inhibition, and a nodule early senescence-like response—may be caused by T3SS effectors of *B. elkanii* USDA61. *L. japonicus* Gifu has all three responses, *L. burttii* induces nodule maturation inhibition and nodule early senescence, and MG-20 induces nodule early senescence only. Infection inhibition is triggered by NopF; the nodule early senescence-like response is triggered by NopM of *B. elkanii* USDA61. Nodule maturation inhibition is triggered by other T3SS effector protein(s).

In the present study, we performed inoculation tests on wild-type *B. elkanii* USDA61 and the T3SS machinery mutant BErhcJ against three *Lotus* accessions, and found accession-dependent responses triggered by T3SEs: infection inhibition in *L. japonicus* Gifu, nodule maturation inhibition in *L. burttii*, and a nodule early senescence-like response in *L. burttii* and *L. japonicus* MG-20. Although infection inhibition triggered by rhizobial T3SEs has been reported in soybean (Okazaki *et al.*, 2009; Tsukui *et al.*, 2013; Yasuda *et al.*, 2016), we herein found nodulation restrictions triggered by rhizobial T3SEs at the post-infection stage. We identified NopF as a candidate trigger of infection inhibition and NopM as that of a nodule early senescence-like response. As indicated by plant phenotypes, *B. elkanii* and even its T3SS-deficient mutant BErhcJ exhibited a lower nitrogen fixation ability estimated by the plant growth phenotype than *M. japonicum* in combination with *Lotus* accessions (Fig. 1D, E, and F), suggesting that *Lotus* accessions use rhizobial T3SEs as markers of unfavorable rhizobial infection and have multiple checkpoints to eliminate rhizobia. *B. elkanii* strains, including 14k062, were isolated from the *Lotus* accessions grown in the field on which soybean had been cultivated over a three-year period. In the second year of the field experiment, all rhizobia strains isolated from the *Lotus* accessions grown in the same field became *Mesorhizobium* stains (unpublished data). This suggests that once the population of favorable rhizobia increased in the field, *Lotus* accessions may distinguish favorable and unfavorable rhizobium strains by recognizing T3SEs. A hypothetical model of the T3SS-mediated interaction between the *Lotus* accessions and *B. elkanii* USDA61 based on the results of the present study is shown in Fig. 8.

An InterPro scan analysis identified leucine-rich repeats (InterPro ID; IPR001611) and a novel E3 ubiquitin ligase domain (InterPro ID; IPR029487) in *B. elkanii* NopM and a BLASTP analysis showed that its homologs were conserved in *Bradyrhizobium* strains and *Sinorhizobium* strains, but not in *Mesorhizobium* strains (Fig. S7). The E3 ubiquitin ligase activity of NopM of *S. fredii* NGR234 was previously shown to reduce the flg22-triggered accumulation of reactive oxygen species (ROS) in *Nicotiana benthamiana* leaves, and the same NopM increased nodule numbers in *Lablab purpureus* (Xin *et al.*, 2012). These findings imply that the E3 ubiquitin ligase activity of NopM of *S. fredii* NGR234 promotes symbiosis by reducing harmful ROS generation in host roots during nodule maturation or senescence. Since ROS accumulate in senescent nodules (Alesandrini *et al.*, 2003; Cam *et al.*, 2012), USDA61 may use NopM as a positive effector to counteract ROS accumulation during nodule senescence. On the other hand, *Lotus* accessions may detect NopM as a post-infection marker of unfavorable rhizobial infection and induce a nodule early senescence-like response. However, the function of NopM in nodule development has not yet been elucidated in detail.

The NopF protein is identical in different *Bradyrhizobium* species. Two copies of *nopF* genes were identified in the *B. diazoefficiens* USDA110 and USDA122 genomes. One copy (Bll1862 and BD122_09540, respectively) is located on symbiosis island A and the other (Bll8201 and BD122_41920) is located in a genome region highly conserved between *B. diazoefficiens* and *B. japonicum* USDA6 and reported as locus C (Kaneko *et al.*, 2011), in which a single-copy *nopF* gene (BJ6T_88750) is located. *BenopF* is also located in the genome region corresponding to locus C. Conjugal transfer protein genes and the replication protein A gene have been identified in this locus (Kaneko *et al.*, 2011); this implies that locus C is transferred between *Bradyrhizobium* species, similar to the symbiotic island. NopF conservation in *Bradyrhizobium* species, including those with two copies in the genome, indicates a strong selection pressure on this T3SE with possible significance in the life cycle of *Bradyrhizobium.* This conservation feature may also be advantageous for the use of NopF by host plants as a signal molecule for infection inhibition.

In soybean with the *Rj4* allele, *B. elkanii* USDA61 was eliminated in a T3SS-dependent manner, and BEL2-5 was identified as a candidate T3SE triggering infection inhibition (Okazaki *et al.*, 2009; Faruque *et al.*, 2015). BEL2-5, encoded by *Be61_51970*, was identified in our MALDI-TOF-MS/MS analysis of extracellular proteins; however, we did not select it as a candidate T3SE because of the high BErhcJ/wild-type ratio (0.64). The InnB protein was recently identified as the T3SE triggering infection inhibition in mung bean (*V. radiata* cv. KPS1) (Nguyen *et al.*, 2018). The InnB protein is encoded by *Be61_78180*, which we confirmed did not induce infection inhibition in *L. japonicus* Gifu. These results suggest that different T3SEs of *B. elkanii* USDA61—BEL2-5 (BE61_51970) in soybean, InnB (BE61_78180) in mung bean, and NopF (BE61_91540) in *L. japonicus*—are recognized by host plants and induce infection inhibition.

In the present study, we demonstrated not only infection inhibition, but also novel responses triggered by T3SS at the post-infection stage, *i.e*., nodule maturation inhibition and a nodule early senescence-like response, in *Lotus* accessions*.* Although these checkpoints have not been reported as T3SS-triggered reactions, nodule maturation inhibition is a typical phenotype observed in cases of nitrogen fixation deficiency caused by mutations in host plants or rhizobia (Krusell *et al.*, 2005; Kumagai *et al.*, 2007; Daubech *et al.*, 2017). A previous study showed that the *nifA* and *nifH* mutants of *S. meliloti* died prematurely after bacteroid elongation in the host plant (Berrabah *et al.*, 2015), suggesting that the host plant monitors the nitrogen fixation ability of symbionts and punishes those with nitrogen fixation deficiencies. A nodule early senescence-like response has been reported in *L. japonicus* MG-20 inoculated with *Rhizobium etli* CE3, which has a lower nitrogen-fixing ability than *M. japonicum* (Banba *et al.*, 2001). Therefore, these post-infection checkpoints may be conserved for monitoring the nitrogen fixation level of symbionts and T3SE recognition.

The T3SEs involved in nodule maturation inhibition in the *Lotus* accessions remain to be identified; however, some T3SEs that inhibit nodule development have been reported. NopT of *S. fredii* NGR234, a C58 cysteine protease with amino acid sequence similarity to AvrPphB of *P. syringae* pv. *phaseolicola*, reduced its nodule number and nodule dry weight following an inoculation with *Crotalaria juncea* (Dai *et al.*, 2008). NopE of *B. diazoefficiens* USDA110, a T3SE containing two EF-hand–like calcium-binding motifs, reduced nodulation efficiency in *V. radiata* KPS2 (Hempel *et al.*, 2009; Wenzel *et al.*, 2010). Among the 9 candidate T3SEs of *B. elkanii* USDA61 identified in the present study (Table 3), none of the single gene disruptants tested (*BenopF*, *BenopL*, *BenopM*, *BenopP1*, *BenopP2*, or *Be61_78180*) affected the nodule maturation inhibition phenotype in *L. burttii* and MG-20. Thus, a future disruption analysis of the remaining three candidate genes and/or multiple gene disruption may contribute to identifying the T3SE(s) triggering nodule maturation inhibition.

The inoculation of *L. japonicus* Gifu with *M. japonicum* carrying *BenopF*, but not with T3SS-disrupted *M. japonicum* carrying *BenopF* markedly reduced nodule numbers and nodule fresh weights (Fig. 7), suggesting that NopF is produced and secreted by the T3SS of *M. japonicum*. Infection inhibition caused by NopF suppressed the stable symbiont, *M. japonicum*, although one or two mature nodules in each plant were occasionally observed. The presence of NopF alone is sufficient to trigger infection inhibition, implying that this T3SE functions in different rhizobial strains. Although the present results may reflect the functional expression of a T3SE in a different rhizobial genus, the secretion of *B. japonicum* Bll8244 has been reported in *S. fredii* HH103 (Yang *et al.*, 2010). As demonstrated by the introduction of *BenopF* into *M. japonicum*, it may be possible to exchange T3SEs between different rhizobial species. For example, it may be feasible to increase the symbiotic potentials of target strains by introducing NopL of *S. fredii* NGR234, which interferes with host mitogen-activated protein kinase signaling and suppresses defense reactions (Bartsev *et al.*, 2004; Zhang *et al.*, 2011).

The inoculation of *M. japonicum* carrying *BenopM* did not induce early senescence in the *Lotus* accessions tested. Based this result, we propose two hypotheses. The first hypothesis is that the expression timing of *tts* box-regulated genes may differ between *M. japonicum* and *B. elkanii* after infection. Okazaki *et al.* showed that *B. elkanii* USDA61 secreted T3SEs without the addition of genistein (Okazaki *et al.*, 2009), suggesting that the flavonoid signal is not essential for T3SS activation in this strain, and the continuous activity of T3SS in *B. elkanii* USDA61 may be expected at the post-infection stage. While *B. elkanii* constitutively secretes T3SEs, *M. japonicum* did not secrete T3SEs at the post-infection stage due to the strict regulation of *tts* box-regulated genes. Another hypothesis is that NopM may function together with additional *B. elkanii* T3SE(s). In a previous study, T3SS of *S. fredii* NGR234 positively affected symbiosis with *T. vogelii*, and the *nopL* and *nopP* double mutant reduced nodule numbers more than a *nopP* single effector mutant (Skorpil *et al.*, 2005), suggesting that the positive effects of this interaction were induced by at least two T3SEs. The absence of the effects of NopM on the early senescence-like response in *M. japonicum* indicates the requirement for additional *B. elkanii* T3SE(s).

In the present study, we demonstrated that *Lotus* accessions have at least three checkpoints to eliminate *B. elkanii* USDA61, and they are regulated by different T3SEs. In addition to infection inhibition, we revealed that nodule maturation inhibition and a nodule early senescence-like response were triggered by T3SEs at the post-infection stage. The present results indicate that leguminous plants continue to recognize rhizobial T3SEs after intracellular infection and attempt to eliminate unfavorable rhizobial strains. In nature, there are risks to host plants associated with infection by inefficient rhizobia that have low or no nitrogen-fixing ability, but produce host-compatible NFs. The present results suggest that host plants use rhizobial T3SEs to monitor unfavorable rhizobia throughout nodulation.

## Supplementary Material

Supplementary Material

## Figures and Tables

**Table 1.** Bacterial strains used in the present study.

**Bacterial strains**	**Characteristics or sequence**^a^	**Reference or source**
***Bradyrhizobium elkanii***		
USDA61	Wild-type strain, Pol^r^	Keyser^b^
BErhcJ	USDA61 derivative harboring an insertion in the *rhcJ* region; Pol^r^, Km^r^, Tc^r^	Okazaki *et al.*, 2009
BEnopL	USDA61 derivative harboring an insertion in the *nopL* region; Pol^r^, Km^r^, Tc^r^	This study
BEnopP1	USDA61 derivative harboring an insertion in the *nopP1* region; Pol^r^, Km^r^, Tc^r^	This study
BEnopP2	USDA61 derivative harboring an insertion in the *nopP2* region; Pol^r^, Km^r^, Tc^r^	This study
BEnopM	USDA61 derivative harboring an insertion in the *nopM* region; Pol^r^, Km^r^, Tc^r^	This study
BEbe61_78180	USDA61 derivative harboring an insertion in the *BE61_78180* region; Pol^r^, Km^r^, Tc^r^	This study
BEnopF	USDA61 derivative harboring an insertion in the *nopF* region; Pol^r^, Km^r^, Tc^r^	This study
14k062	Field-isolated *B. elkanii* strain; Pol^r^	This study
USDA61-DsRed	DsRed-labeled USDA61; Pol^r^, Sp^r^, Sm^r^	Yasuda *et al.*, 2016
BErhcJ-DsRed	DsRed-labeled BErhcJ; Pol^r^, Km^r^, Tc^r^, Sp^r^, Sm^r^	Yasuda *et al.*, 2016
BEnopF-DsRed	DsRed-labeled BEnopF; Pol^r^, Km^r^, Tc^r^, Sp^r^, Sm^r^	This study
14k062-DsRed	DsRed-labeled 14k062; Pol^r^, Sp^r^, Sm^r^	This study
BEnopF-C	BEnopF derivatives complemented with the plasmid pS18mob-nopF; Pol^r^, Km^r^, Sp^r^, Sm^r^,	This study
BEnopM-C_1 and BEnopM-C_2	BEnopM derivatives complemented with the plasmid Tn5::*nopM*; Pol^r^, Km^r^, Sp^r^, Sm^r^,	This study
***Mesorhizobium japonicum***		
MAFF303099	Wild-type strain; Pm^r^	Saeki and Kouchi, 2000
*M. japonicum*-DsRed	DsRed-labeled MAFF303099; Pm^r^	Maekawa *et al.*, 2009
*M. japonicum-*EV	MAFF303099 carrying pHC60; Pm^r^, Tc^r^	Kindly provided by Dr. Yoshikazu Shimoda, National Agriculture and Food Research Organization, Japan
*M. japonicum-BenopF*	MAFF303099 carrying *BenopF*-integrated pHC60; Pm^r^, Tc^r^	This study
*M. japonicum-BenopM*	MAFF303099 carrying *BenopM*-integrated pHC60; Pm^r^, Tc^r^	This study
DT3S	MAFF303099 derivative with genome deletions at positions 5,157,472 to 5,168,624 (mlr6342 to mlr8765), and inserted Km^r^ cassette; Pm^r^, Km^r^	Okazaki *et al.*, 2010
DT3S-*BenopF*	MAFF303099 DT3S carrying *BenopF*-integrated pHC60; Pm^r^, Km^r^ Tc^r^	This study
***Escherichia coli***		
HB101	*recA*, *hsdR*, *hsdM*, *pro*; Sm^r^	Invitrogen
DH5α	F-, Φ80dlacZΔM15, Δ(lacZYA-argF)U169, deoR, recA1, endA1, hsdR17(rK-, mK+), phoA, supE44, λ-, thi-1, gyrA96, relA1	Toyobo
S17-1	*hsdR*, *pro*, *thi* (RP4-2‍ ‍km::Tn*7* tc::Mu, integrated into the chromosome); Sm^r^, Sp^r^	Simon *et al.*, 1983

^a^ Pol^r^, polymyxin resistant; Km^r^, kanamycin resistant; Sm^r^, streptomycin resistant; Sp^r^, spectinomycin resistant; Pm^r^, phosphomycin resistant; Tc^r^, tetracycline resistant; Ap^r^, ampicillin resistant. ^b^ United States Department of Agriculture, Beltsville, MD.

**Table 2.** Plasmids and primers used in the present study.

**Plasmids and primers**	**Characteristics or sequence**^a^	**Reference or source**
**Plasmids**		
pRK2013	ColE1 replicon carrying RK2 transfer genes; Km^r^, *tra*	Figurski and Helinski, 1979
pSUPSCAKm	Derivative of pSUPPOL2SCA with a kanamycin-resistant gene in the *Dra*I site, *oriT* of RP4; Km^r^, Tc^r^	Okazaki *et al.*, 2009
pS18mob	Derivative of pK18mob with a *aadA* in the position of kanamycin-resistant gene, *oriT* of RP4; Sm^r^, Sp^r^	Okazaki *et al.*, unpublished
pS18mob-nopF	pS18mob carrying a 2.0-kb DNA fragment containing a non-coding region and *nopF* and its upstream *tts* box region; Sp^r^, Sm^r^	This study
pBjGroEL4::DsRed2	DsRed transposon delivery vector; Sp^r^, Sm^r^, Ap^r^	Hayashi *et al.*, 2014
Tn5::nopM	pBjGroEL4::DsRed2 carrying a 2.9-kb DNA fragment containing *nopM* and its upstream *tts* box region; Sp^r^, Sm^r^, Ap^r^	This study
pHC60	GFP constitutively-expressing vector; Tc^r^	Cheng and Walker, 1998
pHC60-*BenopM*	pHC60 carrying *BenopM* and its upstream *tts* box promotor; Tc^r^	This study
pHC60-*BenopF*	pHC60 carrying *BenopF* and its upstream *tts* box promotor; Tc^r^	This study
**Primers**		
nopL_F	5′-ACCGCGGTGGCGGCCAACTCAATCAGCCCAACG-3′	This study
nopL_R	5′-CGGGGGATCCACTAGTATGAAACGCTCGTCCTCGG-3′	This study
nopP1_F	5′-ACCGCGGTGGCGGCCTATTCCCTCGTGACCAAGCC-3′	This study
nopP1_R	5′-CGGGGGATCCACTAGCGCTATTCGTTGTCCATTTG-3′	This study
nopP2_F	5′-ACCGCGGTGGCGGCCATCGCTCTTCCTTCAATGAC-3′	This study
nopP2_R	5′-CGGGGGATCCACTAGTATCACCATCCCCTGCCTTG-3′	This study
nopM_F	5′-ACCGCGGTGGCGGCCGCACTCCTTCGGGAACTTC-3′	This study
nopM_R	5′-CGGGGGATCCACTAGAGGTCGGGCAGATTGGTC-3′	This study
BE61_78180int-NotI-L	5′-ACGAAGCGGCCGCGAGAGTTCCGCAAAGTCGAG-3′	This study
BE61_78180int-SpeI-R	5′-TATCTACTAGTCAATTGAGGGCCTATCGTTG-3′	This study
nopFint-NotI-L	5′-ACGAAGCGGCCGCAGGTGTGTCAGTCCGCCTAC-3′	This study
nopFint-SpeI-R	5′-TATCTACTAGTAAATGACAGTCCGCATTTCC-3′	This study
nopM-NotI-L	5′-ATTAAGCGGCCGCTCAGAATAGGTGGGGACTCG-3′	This study
nopM-NotI-R	5′-TATCTGCGGCCGCTTTCCTTCACCGGGTATCTG-3′	This study
nopM-SacI-L	5′-ACGTCGAGCTCTCAGAATAGGTGGGGACTCG-3′	This study
nopM-SacI-R	5′-ATTGCGAGCTCTTTCCTTCACCGGGTATCTG-3′	This study
nopF-NotI-L	5′-ATTAAGCGGCCGCGTAAAGGACCGGCTCATGC-3′	This study
nopF-NotI-R	5′-TATCTGCGGCCGCCCCTCAGGCGCACTCTTAC-3′	This study
pS18mob_EcoR1_inf	5′-CCATGATTACGAATTGATTTGGAATTGCGCTTGAT-3′	This study
nopF_inf_1	5′-GAGCCGGTCCTTTACTTGATGAGCCTGATGTGAG-3′	This study
nopF_inf_2	5′-GTAAAGGACCGGCTCATG-3′	This study
nopF_inf_3	5′-TACCGAGCTCGAATTCCCTCAGGCGCACTCTTA-3′	This study
nopF_out_F	5′-CAGATGGTGCTGCTTTTACG-3′	This study
nopF_out_R	5′-CTCCATCTCGCCCATAAGAA-3′	This study
nopM_out_F	5′-TCAGAATAGGTGGGGACTCG-3′	This study
nopM_out_R	5′-TTTCCTTCACCGGGTATCTG-3′	This study

^a^ Km^r^, kanamycin resistant; Sm^r^, streptomycin resistant; Sp^r^, spectinomycin resistant; Pm^r^, phosphomycin resistant; Tc^r^, tetracycline resistant; Ap^r^, ampicillin resistant.

**Table 3.** Extracellular protein analysis using the iTRAQ system.

Accession	Description	Molecular weight (kDa)	Total prot score	Coverage (%)ª	Peptides^b^	Fold change^c^ BErhcJ/WT
BE61_80730	Nodulation outer protein NopP	31.0	23.39	62.2	13	0.02
BE61_78180	Unknown protein	83.0	22.21	27.8	11	0.15
BE61_80150	Nodulation outer protein NopX	63.4	19.44	31.1	10	0.02
BE61_80320	Nodulation outer protein NopM	67.0	18.51	29.2	10	0.12
BE61_77110	Nodulation outer protein NopP	31.4	17.14	39.9	10	0.05
BE61_76200	Unknown protein	48.6	15.10	33.0	8	0.08
BE61_80180	Nodulation outer protein NopA	7.5	14.72	93.5	7	0.01
BE61_91540	Unknown protein (NopF)	19.2	8.01	45.3	4	0.02
BE61_78310	Unknown protein	19.9	8.00	25.0	3	0.04
BE61_51850	Unknown protein	33.1	7.72	30.8	3	0.11
BE61_80070	Nodulation outer protein NopL	24.6	6.16	29.7	3	0.01

^a^ Sequence coverage. ^b^ The total number of detected peptides (at the 95% confidence level) for each protein. ^c^ Fold changes in the T3SS mutant BErhcJ vs. wild-type USDA61.
