# Supplementary material for: Lotus Accessions Possess Multiple Checkpoints Triggered by Different Type III Secretion System Effectors of the Wide-Host-Range Symbiont Bradyrhizobium elkanii USDA61
Source: Microbes Environ. 2020 Feb 20;35(1):ME19141. doi: 10.1264/jsme2.ME19141 (PMC7104275; doi:10.1264/jsme2.ME19141)
Supplement: Supplementary file 1 — Supplementary Material [file 35_19141_s1.pdf]

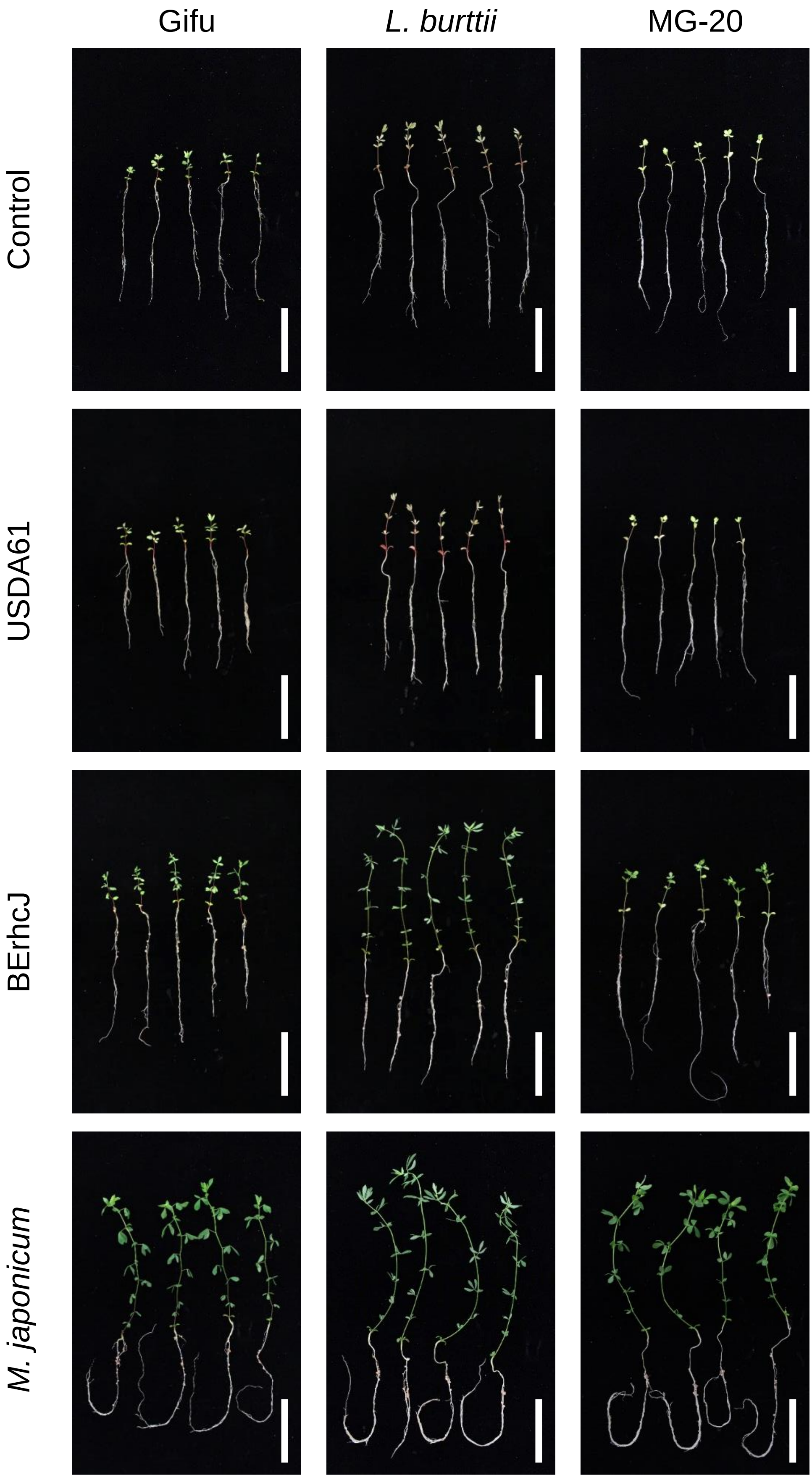

Fig. S1 Growth phenotype of *Lotus* accessions inoculated with *B. elkanii* strains or *M. japonicum*. Plants inoculated with *B. elkanii* USDA61, T3SS-deficient mutant BErhcJ, or *M. japonicum* MAFF303099 and no inoculation control are shown at 30 days post inoculation. Scale bars = 5 cm.

Fig. S1 Kusakabe et al.

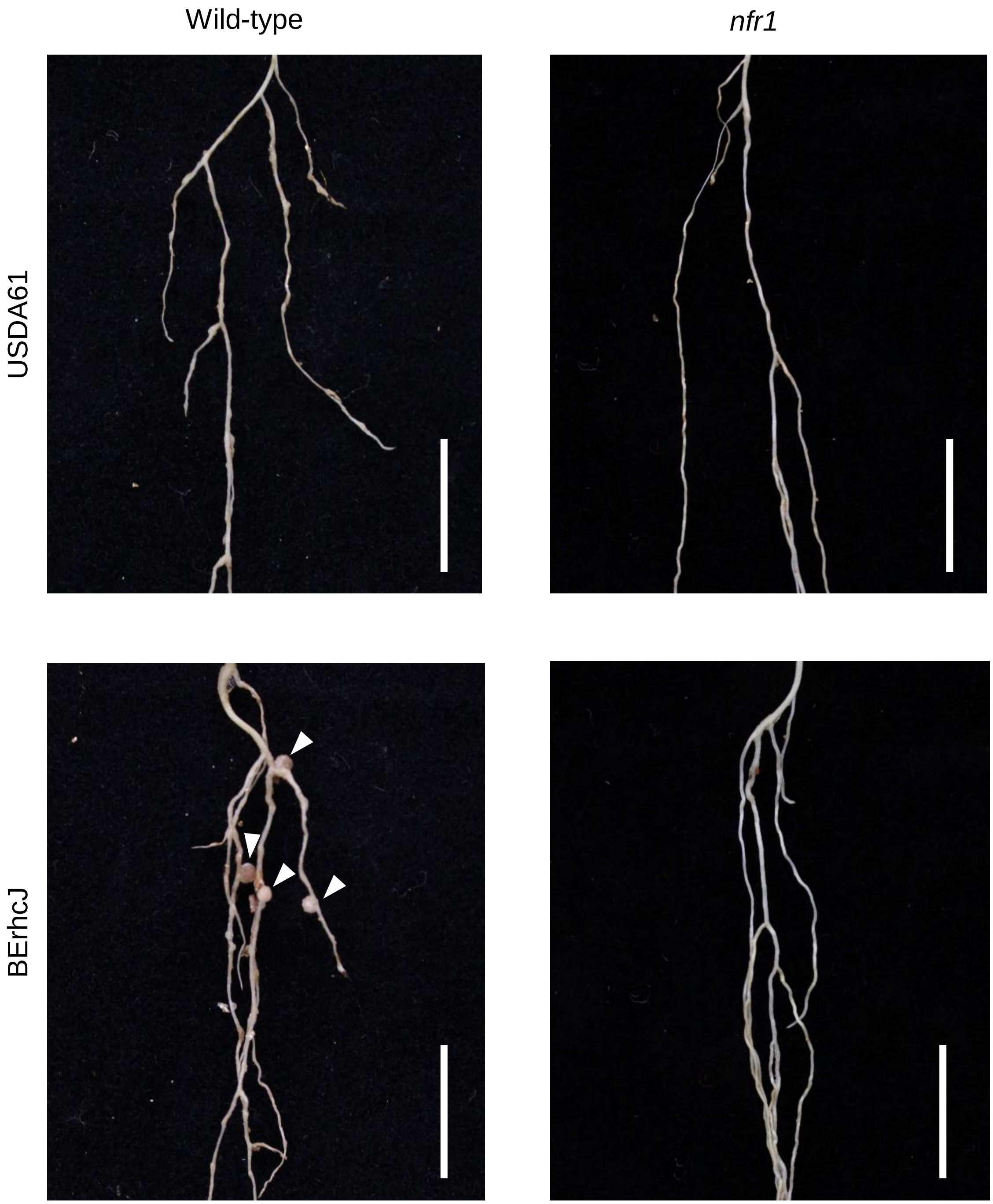

Fig. S2 Inoculation test of *B. elkanii* USDA61 and the T3SS mutant in the *L. japonicus* nod factor receptor 1 mutant (*nfr1*). Wild-type *L. japonicus* Gifu and *nfr1* were inoculated with wild-type *B. elkanii* USDA61 or T3SS-deficient mutant BErhcJ, and the roots were observed at 30 days post inoculation. Arrowheads indicate mature nodules. Scale bars = 1 cm.

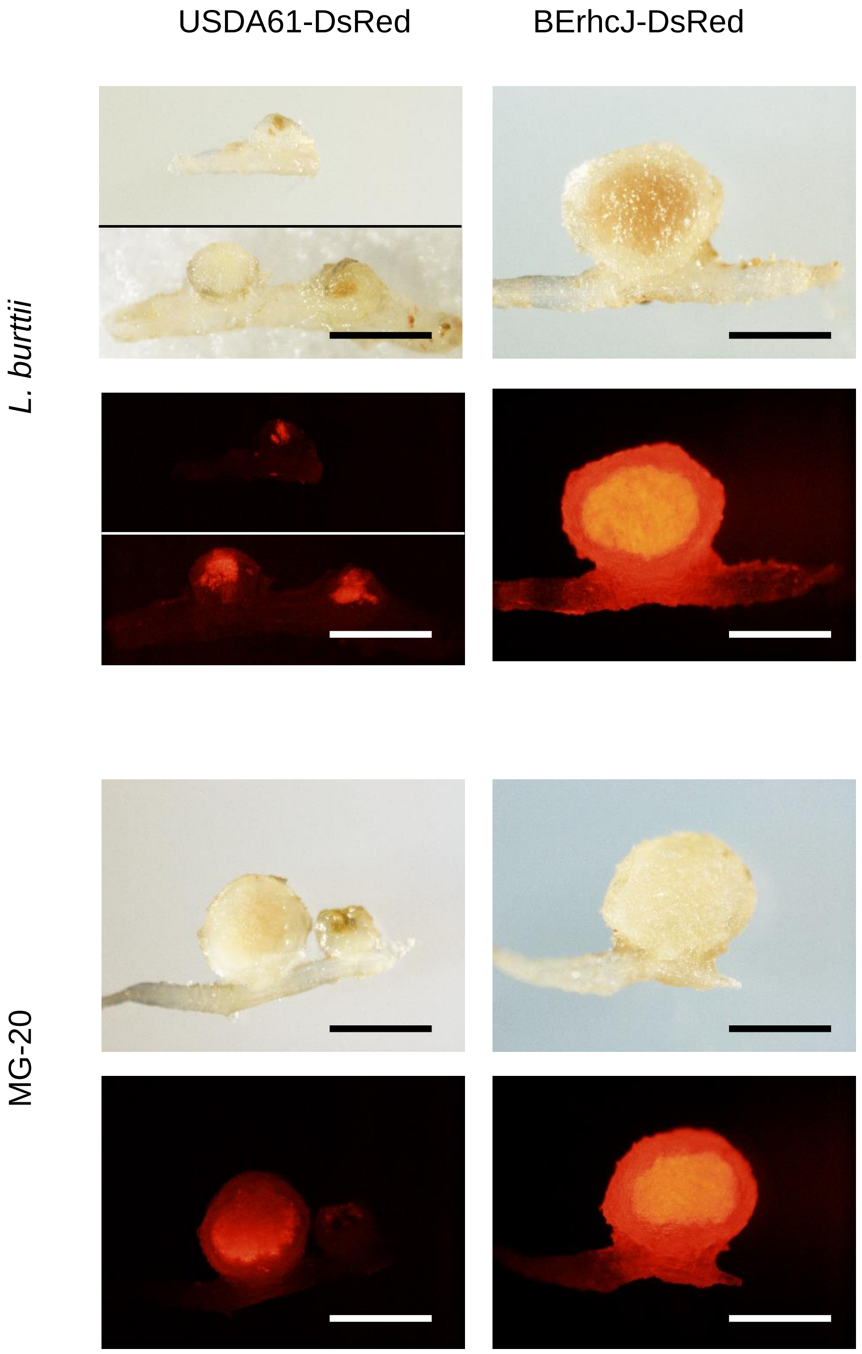

Fig. S3 Bright-filed images (top) and DsRed-filtered fluorescence images (bottom) of sections of nodules on *L. burttii* and MG-20 infected by DsRed-labeled USDA61 and BErhcJ. Nodule sections were observed at 30 days post inoculation. Scale bar = 1 mm.

Fig. S3 Kusakabe et al.

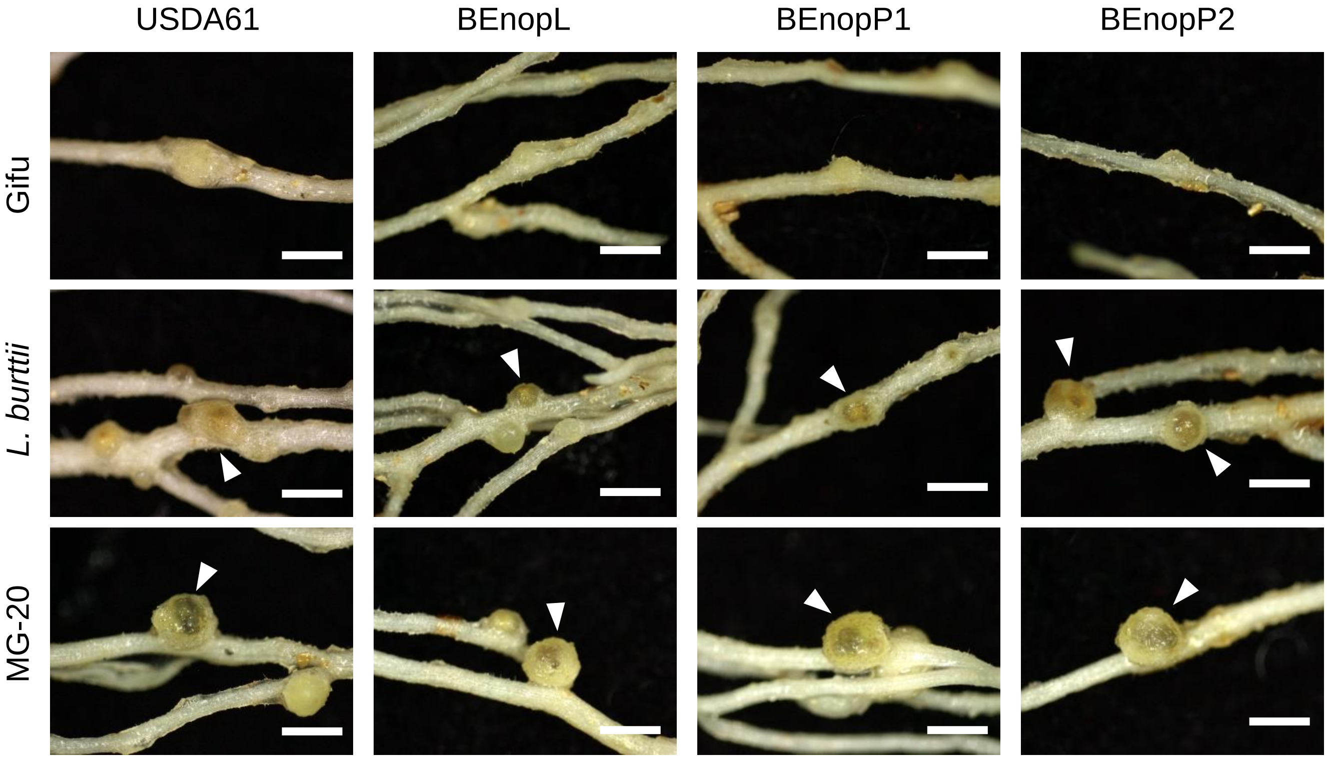

Fig. S4 Symbiotic phenotypes of *Lotus* accessions inoculated with the mutants of *nopL*, *nopP1*, or *nopP2*. *Lotus japonicus* Gifu, *L. burttii*, and *L. japonicus* MG-20 were inoculated with *Bradyrhizobium elkanii* USDA61 or the mutants of *BenopL* (BEnopL), *BenopP1* (BEnopP1), or *BenopP2* (BEnopP2), and nodules were observed at 30 days post inoculation. Arrowheads indicate brownish nodules. Scale bars = 1 mm.

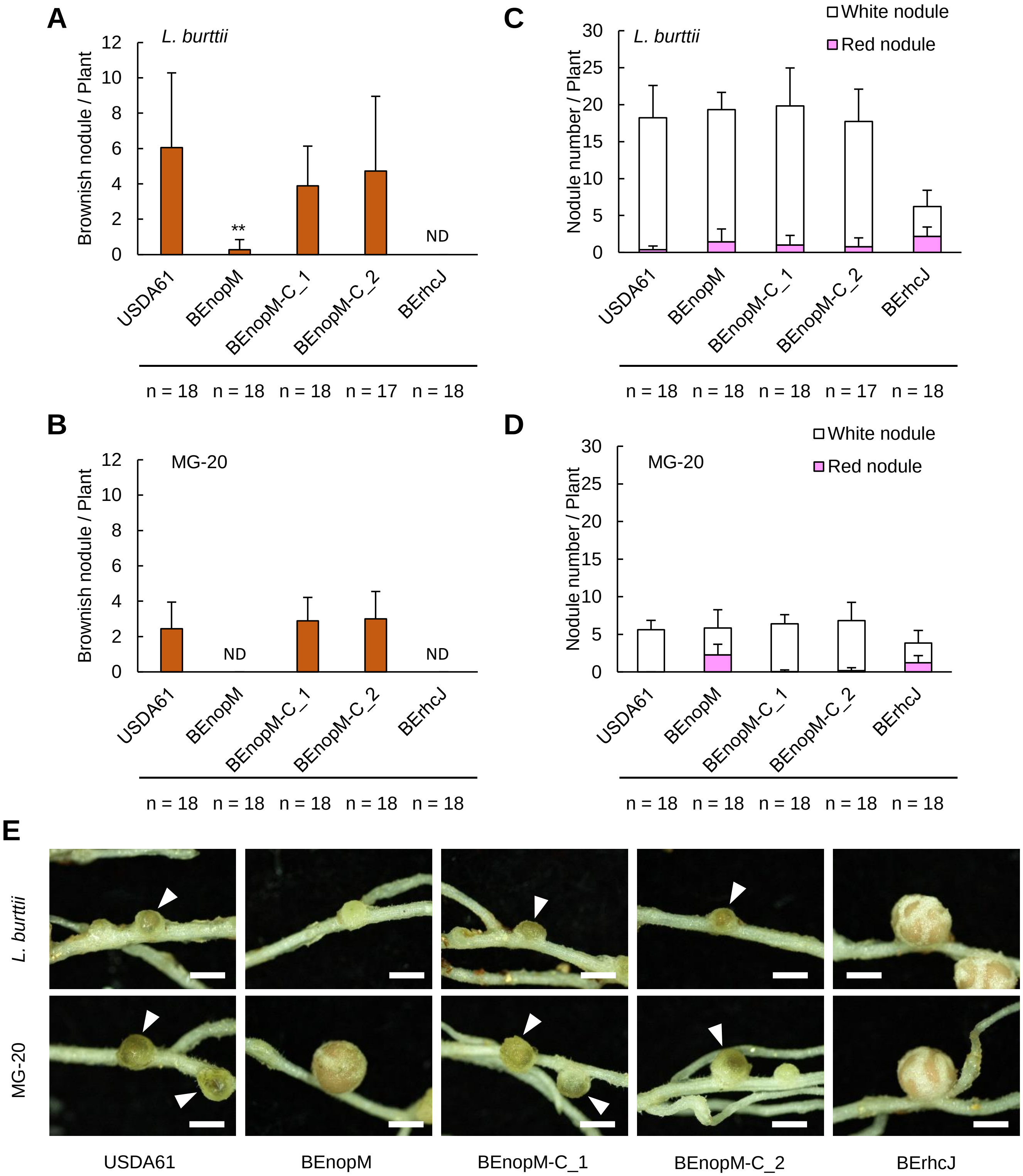

Fig. S5 Kusakabe et al.

Fig. S5 Complementation test of the *BenopM* mutant. Phenotypes of *L. burttii* (A, C) and *L. japonicus* MG-20 (B, D) inoculated with wild-type *B. elkanii* USDA61, *BenopM* mutant (BEnopM), two independent *nopM*-complemented strains derived from BEnopM (BEnopM-C_1 and BEnopM-C_2), or T3SS-deficient mutant (BErhcJ) were analyzed at 30 days post inoculation. (A, B) Number of brownish nodules and (C, D) total number of mature and white nodules on the roots of (A, C) *L. burttii* and (B, D) MG-20 are shown. ND means not detected. In panel C and D, brownish nodules were included in the count of white nodules. Inoculation tests were performed three times with 5 or 6 plants each time. Error bars indicate standard deviations. Student's *t*-test was performed for brownish nodule counts; ** $P < 0.01$ vs. wild-type USDA61. (E) Root nodules of two *Lotus* accessions inoculated with the above bacteria at 30 days post inoculation. Scale bars = 1 mm.

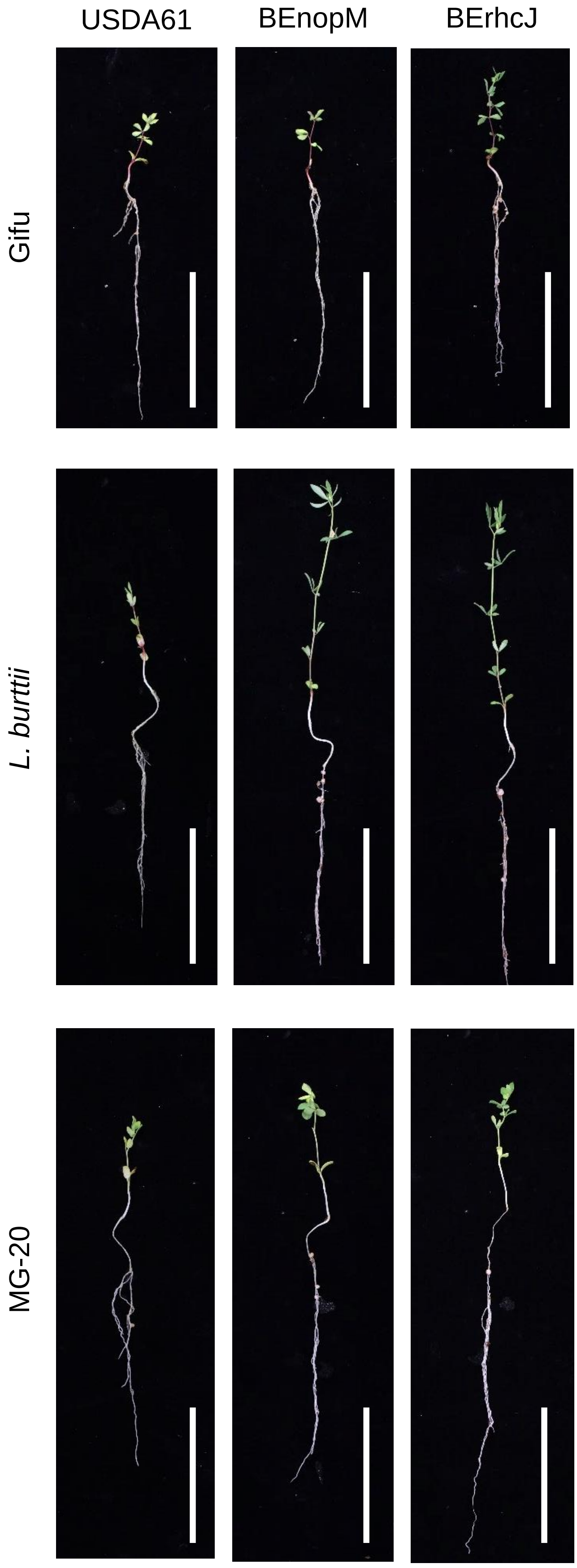

Fig. S6 Growth phenotypes of *Lotus* accessions inoculated with the *BenopM* mutant. Plants were imaged at 30 days post inoculation. Scale bars = 5 cm.

Fig. S6 Kusakabe et al.

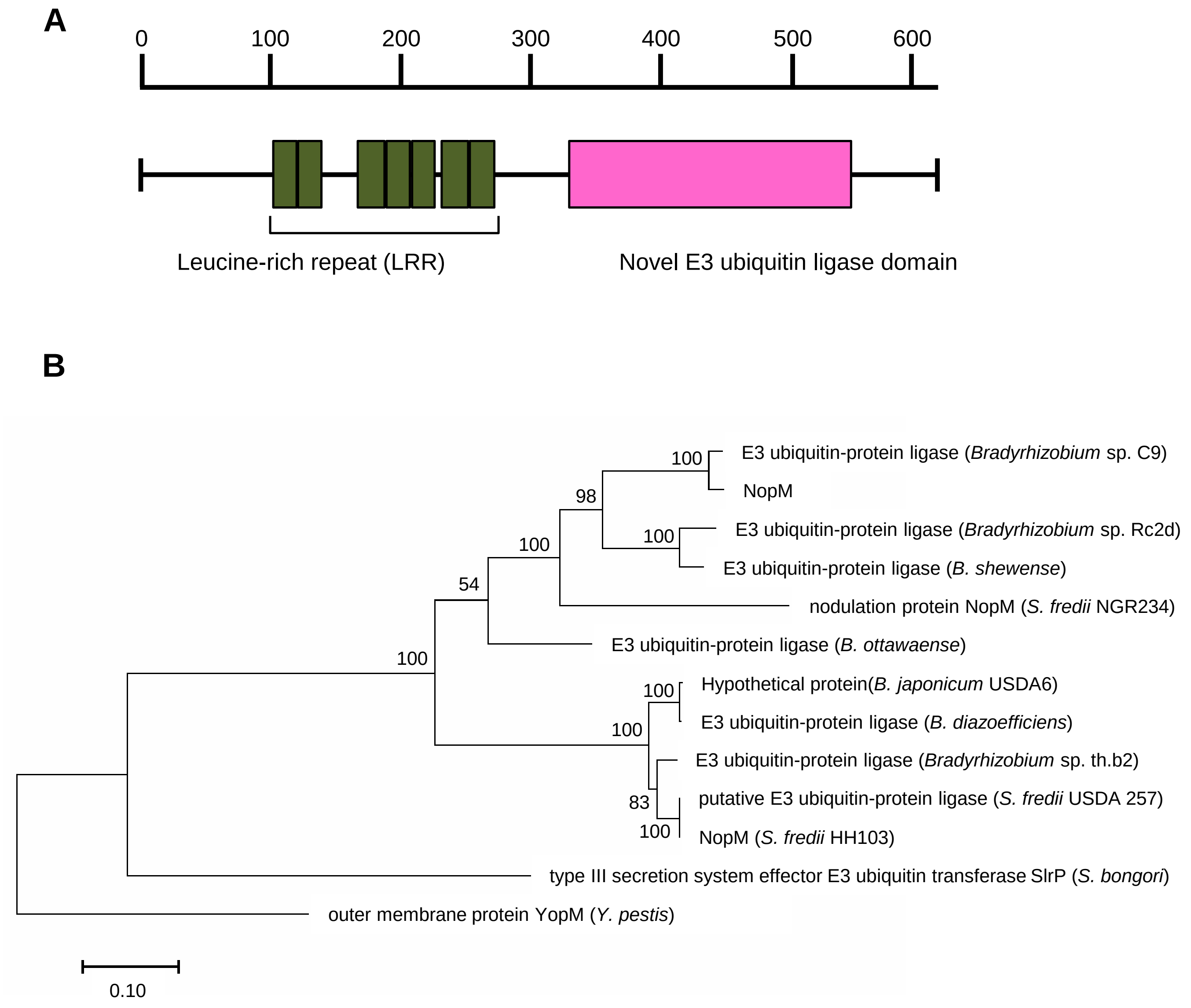

Fig. S7 Kusakabe et al.

Fig. S7 Domain organization and phylogenetic analysis of NopM proteins. (A) Structural features of NopM in *B. elkanii* USDA61 (610 amino acids; accession number LC471585). Green boxes, leucine-rich repeats (IPR001611, residues 104–124, 125–146, 166–186, 187–208, 209–226, 228–248, and 249–270); pink box, a novel E3 ligase domain (IPR029487, residues 331–542). (B) Phylogenetic tree of bacterial NopM homologues. Amino acid sequences were aligned in the ClustalW program (http://clustalw.ddbj.nig.ac.jp) and the phylogenetic tree was constructed in MEGA7 software (Kumar *et al.*, 2016). Percentage of bootstrap support (out of 1000 replicates) is indicated at each tree node (only if >50%). GenBank accession numbers are as follows: outer membrane protein YopM of *Yersinia pestis*, WP_002224885.1; type III secretion system effector E3 ubiquitin transferase SlrP of *Salmonella bongori*, WP_000482016.1; E3 ubiquitin-protein ligase of *Bradyrhizobium* sp. C9, WP_097671487.1; E3 ubiquitin-protein ligase of *Bradyrhizobium* sp. Rc2d, (WP_091881754.1; E3 ubiquitin-protein ligase of *Bradyrhizobium shewense*, WP_091956592.1; nodulation protein NopM of *Sinorhizobium fredii* NGR234, NP_443862.1; E3 ubiquitin-protein ligase of *Bradyrhizobium ottawaense*, WP_097669299.1; hypothetical protein BJ6T_78970 of *Bradyrhizobium japonicum* USDA6, BAL13143.1; E3 ubiquitin-protein ligase of *Bradyrhizobium diazoefficiens*, WP_038968052.1; putative NopM of *S. fredii* HH103, CCC55885.1; putative E3 ubiquitin-protein ligase of *S. fredii* USDA257, AFL55168.1; and E3 ubiquitin-protein ligase of *Bradyrhizobium* sp. th.b2, WP_035978391.1. Scale bar represents 0.1 substitutions per site.

Fig. S7 continued Kusakabe et al.

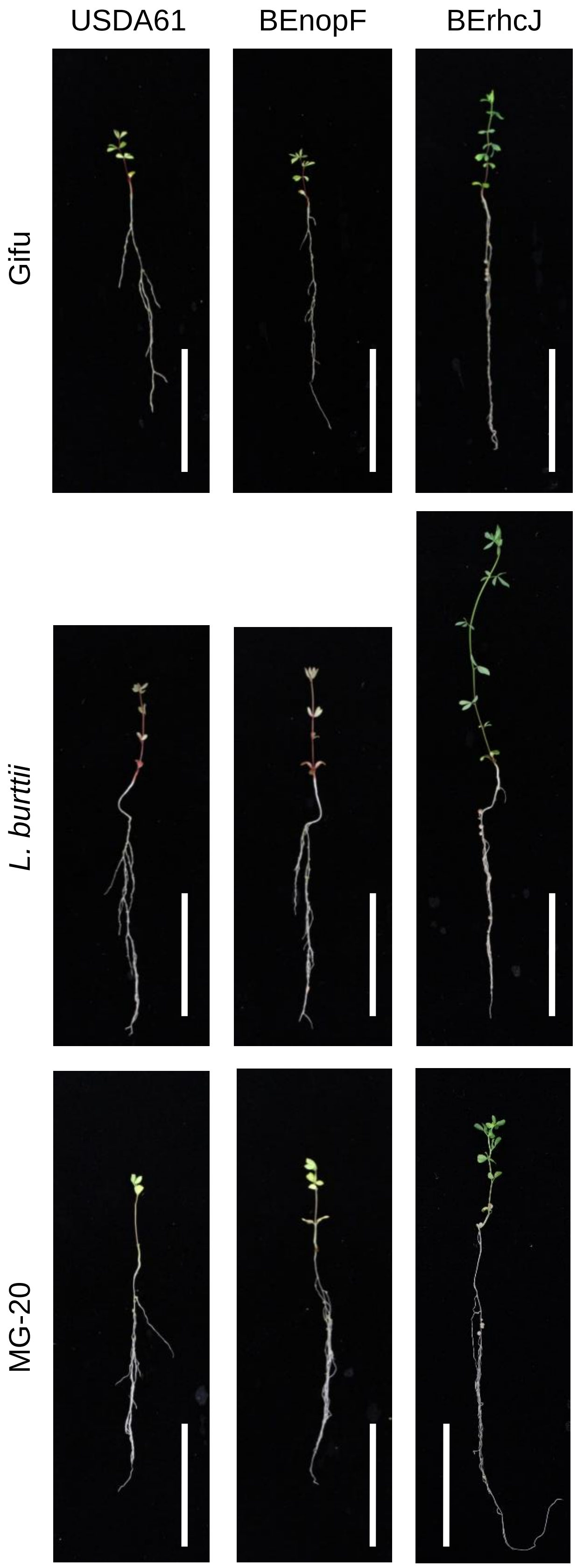

Fig. S8 Growth phenotypes of *Lotus* accessions inoculated with the *nopF* and T3SS mutants of *B. elkanii* USDA61. Plants were imaged at 30 days post inoculation. Scale bars = 5 cm.

Fig. S8 Kusakabe et al.

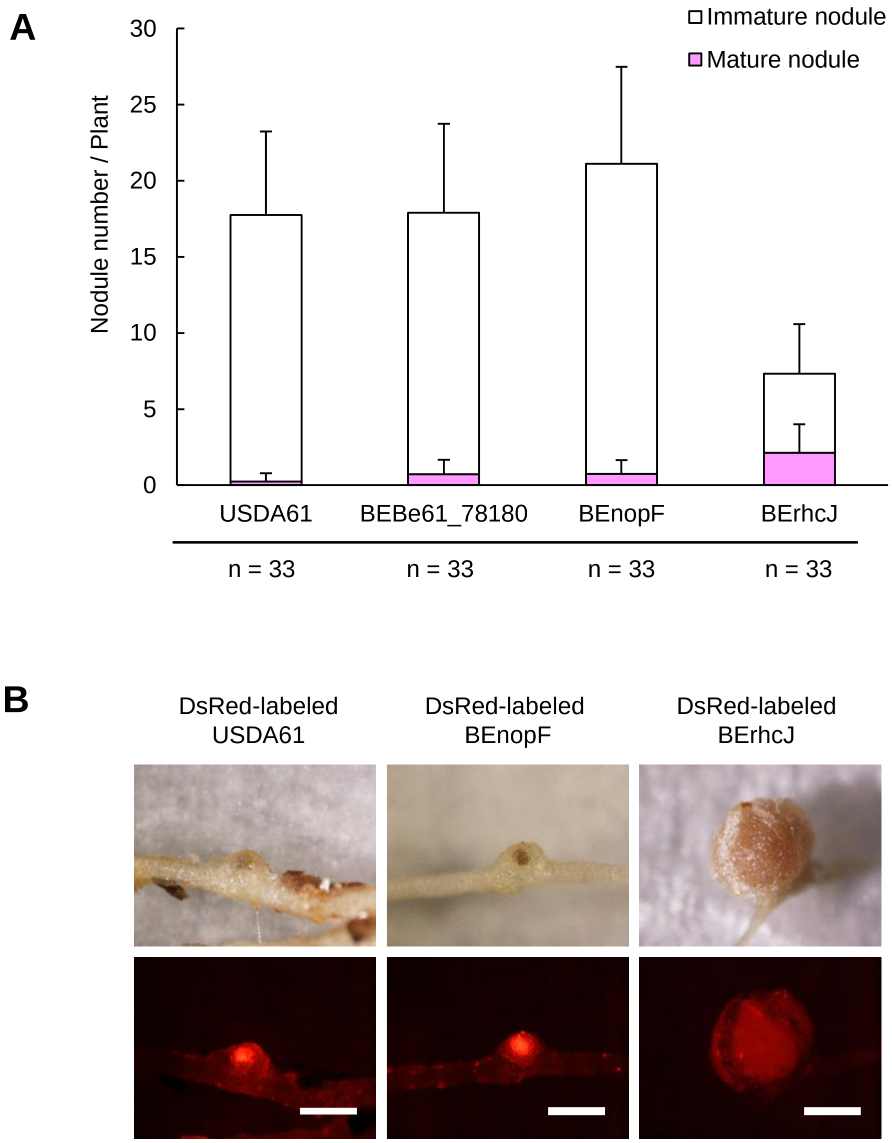

Fig. S9 Effect of NopF on nodule formation in *L. burttii*. (A) Nodule number at 30 days post inoculation with wild-type *Bradyrhizobium elkanii* USDA61, *Be61_78180* mutant (BEbe61_78180), *nopF* mutant (BEnopF), or T3SS-deficient mutant (BErhcJ). Inoculation tests were performed twice with 9 to 12 plants each time. Error bars indicate standard deviations. (B) Infection phenotype of *L. burttii* inoculated with the indicated DsRed-labeled strains. DsRed fluorescence was observed under a fluorescence microscope at 30 days post inoculation. Scale bars = 1 mm.

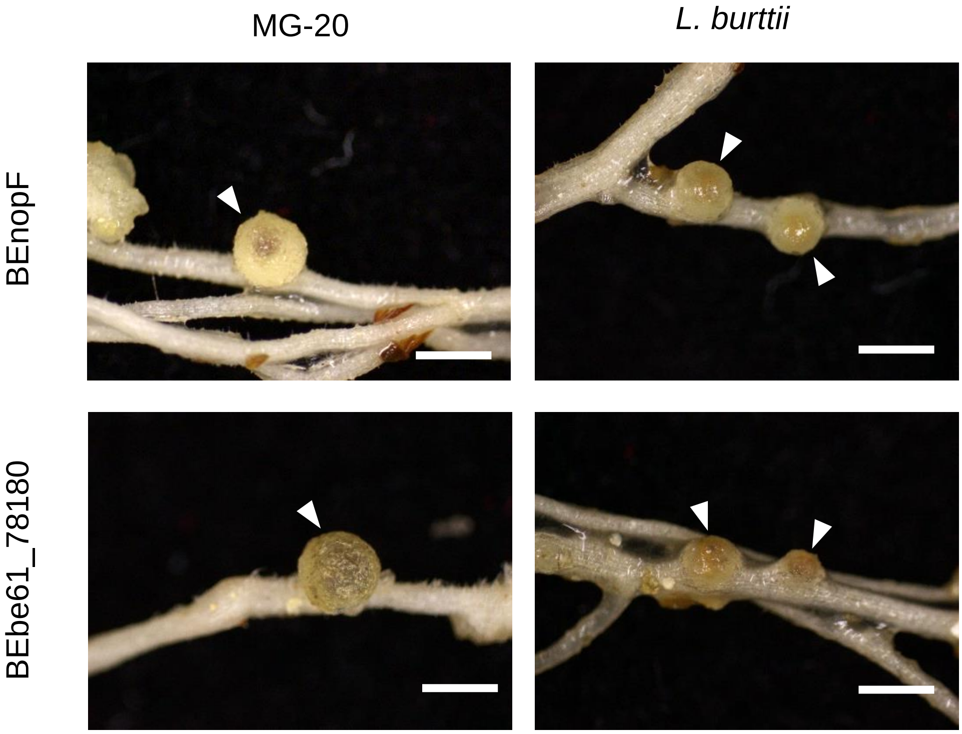

Fig. S10 Nodule phenotypes of *L. japonicus* MG-20 and *L. burttii* inoculated with the *BenopF* and *Be61_78180* mutants. Brownish nodules (arrowheads) are shown at 30 days post inoculation. Scale bars = 1 mm.

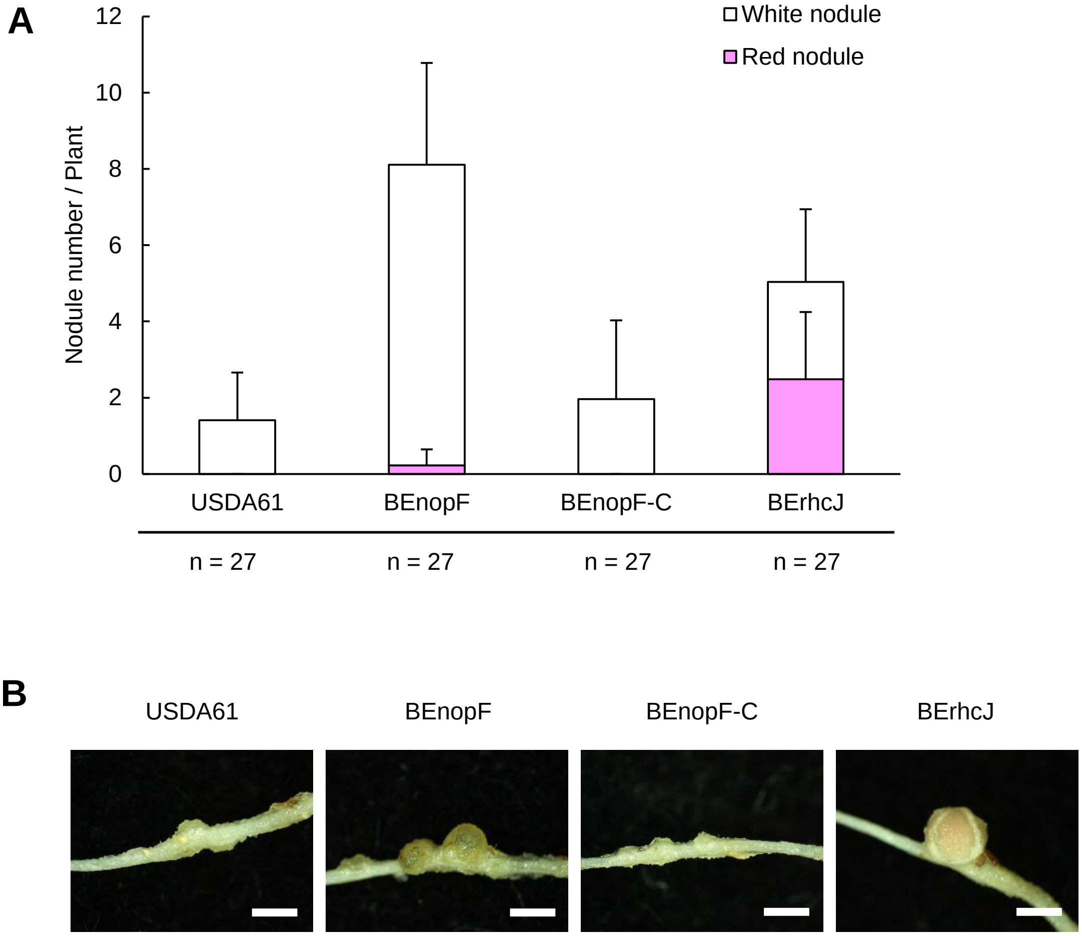

Fig. S11 Complementation test of the *BenopF* mutant on *L. japonicus* Gifu. (A) Nodule number after inoculation with wild-type *B. elkanii* USDA61, *BenopF* mutant (BEnopF), *BenopF*-complemented strain derived from BEnopF (BEnopF-C), or T3SS-deficient mutant (BErhcJ) at 30 days post inoculation. Inoculation tests were performed three times with 9 plants each time. Error bars indicate standard deviations. (B) Nodule phenotype of plants inoculated with the above bacteria at 30 days post inoculation. Scale bars = 1 mm.

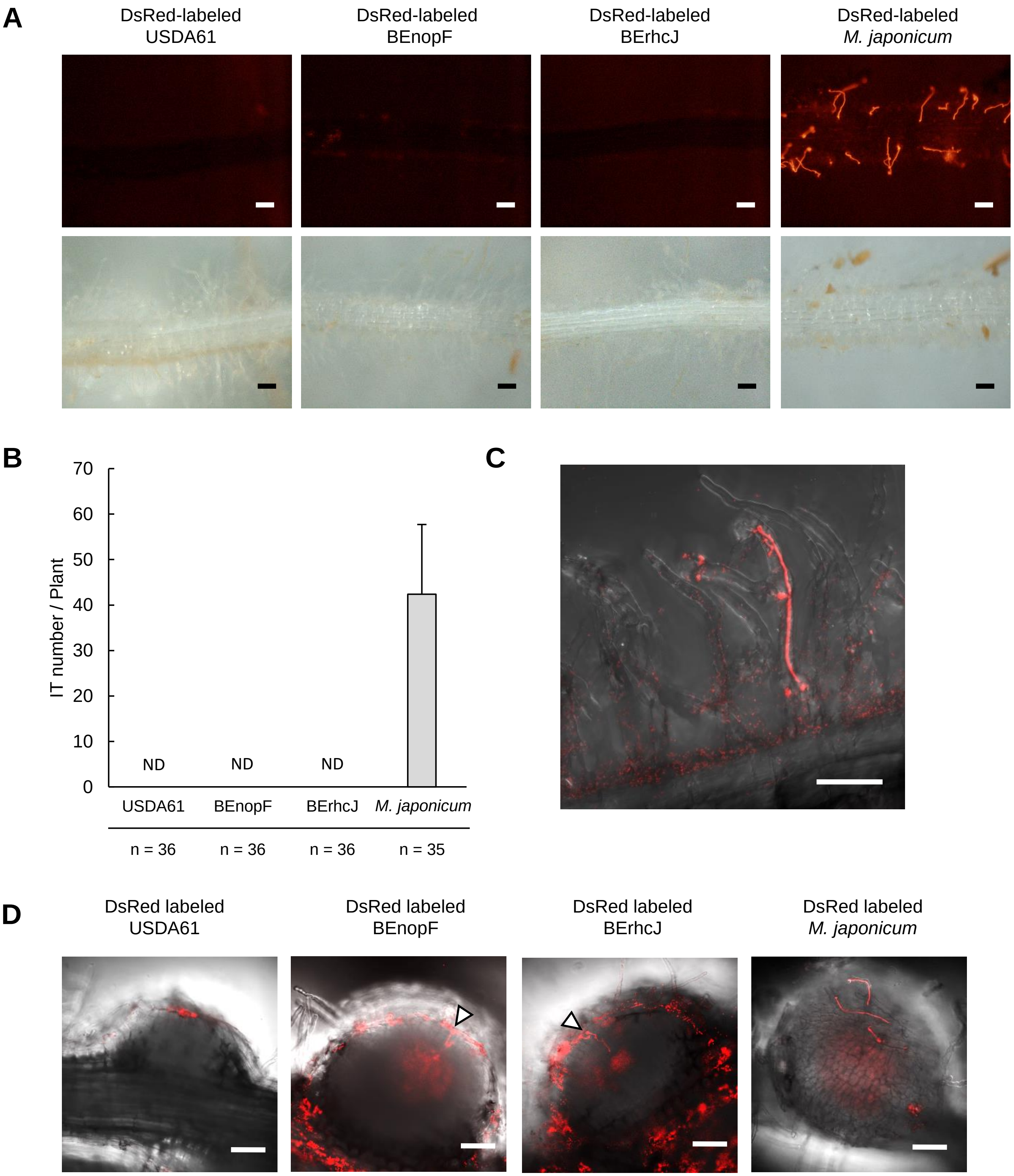

Fig. S12 Kusakabe et al.

Fig. S12 Infection phenotypes of *L. japonicus* Gifu inoculated with *B. elkanii* strains or *M. japonicum*. (A) Images and (B) numbers of infection threads (ITs) at 10 days post inoculation with DsRed-labeled rhizobial strains. Scale bars = 100 µm. (C) Close-up image of ITs after inoculation with *M. japonicum* MAFF303099. Scale bar = 50 µm. (D) Confocal images of the nodule surface at 14 days post inoculation with DsRed-labeled rhizobial strains. Arrowheads indicate putative crack entry points. Scale bars = 100 µm.

A

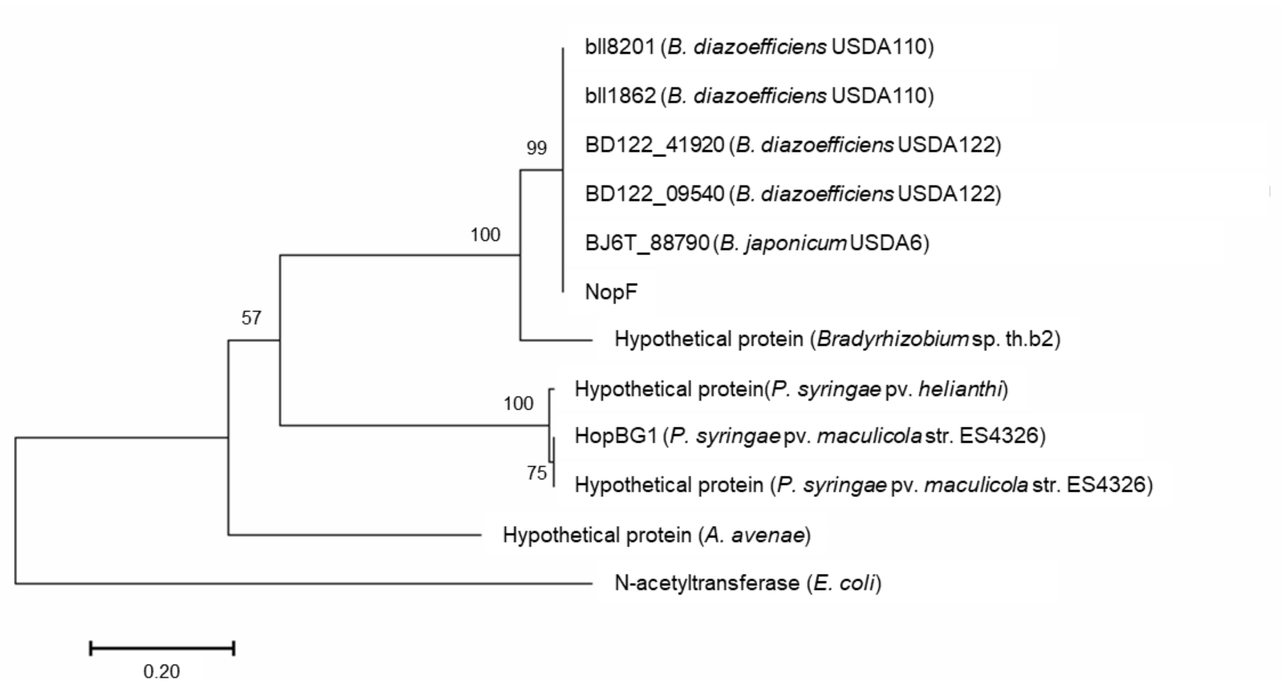

B

```
  NopF     1   MRTNYNNWPSDGQSVDQQRDALAGTSGADGAAFTAALAAGPASSAFDKRT   50
HopBG1     1   -----------------------------------------MQIYDRGR   8

  NopF    51   GVSVRLHRVSSNIGNAQSVSLFTDANERNK-VGAMTVAPGRNSLRILSIE   99
HopBG1     9   QSMIDVAIESSASGNAKKIEIRSASPTRSQHFGTMSVAPQLNSLRLLSIE   58

  NopF   100   NLGRSRYKGVGTAMIEVADHTRQSAGLSKLSLLSQDEGASAFFYKKGFRF   149
HopBG1    59   NHKKTAVRQVGSRFLEIADRMRSDLALSSVSLMCQDEGAAKFFYKNGFRF   108

  NopF   150   ADEG---KNAEMRTVISNPR-YVSDEIL-MGEMER-                179
HopBG1   109   VGSGADAKNSALKHHIDHPQDALPDEIVFLGDMERK                144
```

Fig. S13 Kusakabe et al.

Fig. S13 Phylogenetic analysis and structural features of NopF. (A) Phylogenetic tree of bacterial NopF homologues. Amino acid sequences were aligned in the ClustalW program (http://clustalw.ddbj.nig.ac.jp) and a phylogenetic tree was constructed in MEGA7 software (Kumar *et al*., 2016). Percentage of bootstrap support (out of 1000 replicates) is indicated at each tree node (only if >50%). GenBank accession numbers are as follows: NopF (BE61_91540) of *B. elkanii* USDA61, LC471586; N-acetyltransferase of *Escherichia coli*, WP_103281195.1; HopBG1 of *Pseudomonas syringae* pv. *maculicola* str. ES4326, ADQ74901.1; hypothetical protein of *P. syringae* pv. *helianthi*), KPX46812.1; hypothetical protein of *P. syringae* pv. *maculicola* str. ES4326, EGH63103.1; hypothetical protein of *Acidovorax avenae*, WP_019699793.1; hypothetical protein of *Bradyrhizobium* sp. th.b2, WP_029085180.1; bll8201 of *Bradyrhizobium diazoefficiens* USDA110, BAC53466.1; bll1862 of *B. diazoefficiens* USDA110, BAC47127.1; BD122_41920 of *B. diazoefficiens* USDA122, APO56996.1; BD122_09540 of *B. diazoefficiens* USDA122, APO50480.1; and BJ6T_88790 of *Bradyrhizobium japonicum* USDA6, BAL14121.1. Scale bar represents 0.2 substitutions per site. (B) Amino acid sequence alignment of NopF in *Bradyrhizobium* species and HopBG1 of *P. syringae* pv. *maculicola* str. ES4326. Identical amino acid residues are shown in red.

B. elkanii USDA61	-	TTGTCAGCTTTCTGTCAGCTCGTCCAAATA	-	13 bp	-	*nopF*
B. elkanii USDA61	-	CCGTCAGCTTATCGACAGCTACGTCTCGTA	-	282 bp	-	*nopM*
B. elkanii USDA61	-	TCGTCAGCTTTTCGAAAGCTAGGTCCCATA	-	95 bp	-	*BE61_78180*
M. japonicum MAFF303099	-	TCGTCAGTTTACCGAAAGCTAAACCGCTCA	-	100 bp	-	*nopX*
M. japonicum MAFF303099	-	CCGTCAGCTAATCGTCAGCCAAGCGATCTA	-	218 bp	-	*mlr6361*
tts box consensus	-	tcGTCAGctT.tcGaaAGct...cc.cctA	-			

Fig. S14 Conservation of the *tts* box sequence in *B. elkanii* and *M. japonicum*. The *tts* box sequences of *nopF*, *nopM*, and *Be61_78180* of *B. elkanii* USDA61, and *nopX* and *mlr6361* of *M. japonicum* MAFF303099 are shown. The *nopX* and *mlr6361* genes of *M. japonicum* MAFF303099 are TtsI-inducible, and Mlr6361 is reported to be a T3SS effector protein (Okazaki *et al*., 2010). In the consensus sequence, all invariant nucleotides are capitalized and lowercase letters are used for nucleotides conserved in at least 50% of the analyzed sequences (Krause *et al*., 2002). Nucleotides in common with the consensus sequence in all five sequences shown are in red.

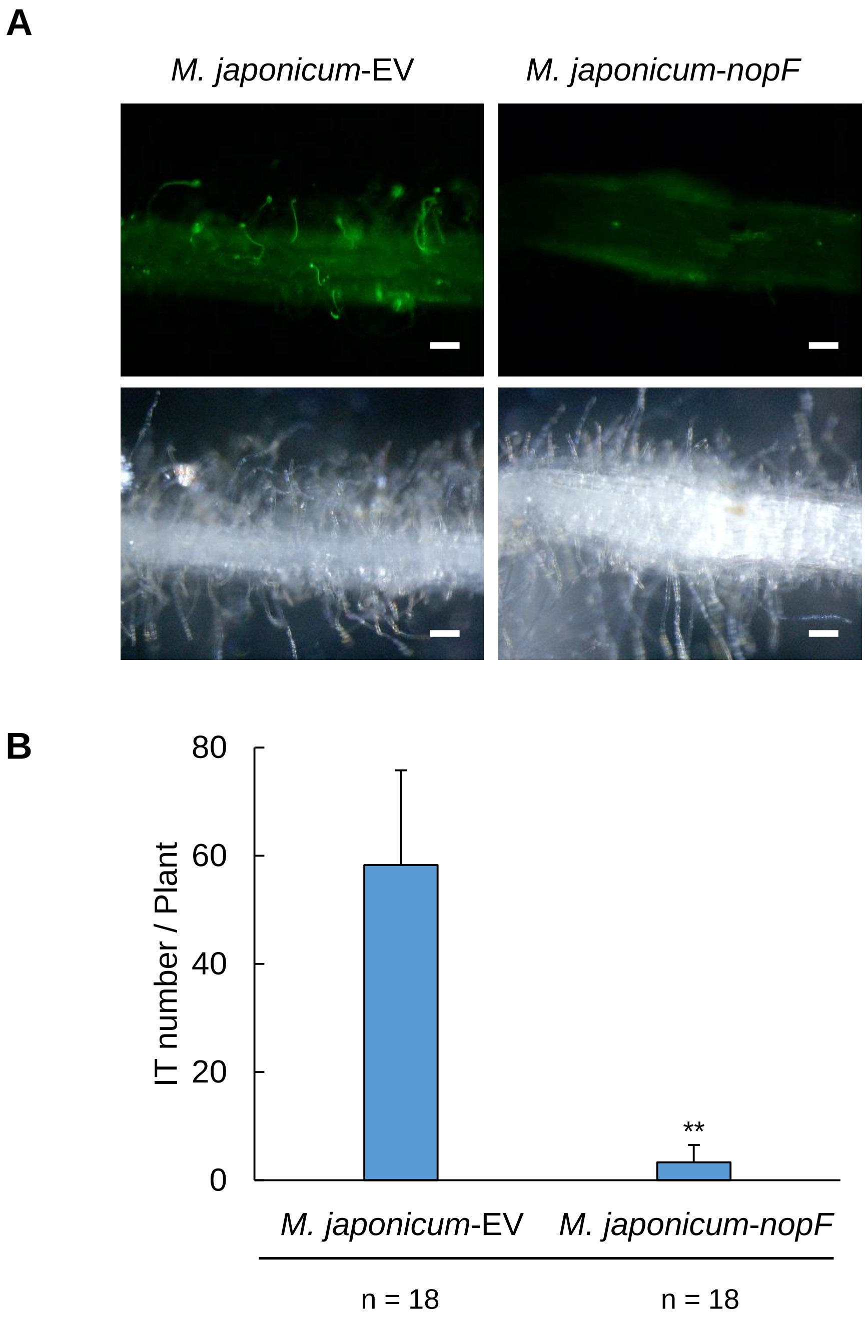

Fig. S15 Infection phenotypes of *L. japonicus* Gifu inoculated with *M. japonicum*-EV or *M. japonicum-nopF*. GFP-filtered fluorescence images (A-top), bright-filed images (A-bottom), and numbers of infection threads (B) at 10 days post inoculation with GFP-labeled rhizobial strains. Inoculation tests were performed three times with 6 plants each time. Error bars indicate standard deviations. ** $P < 0.01$ vs. *M. japonicum*-EV in Student's *t* test. Scale bars = 100 µm.

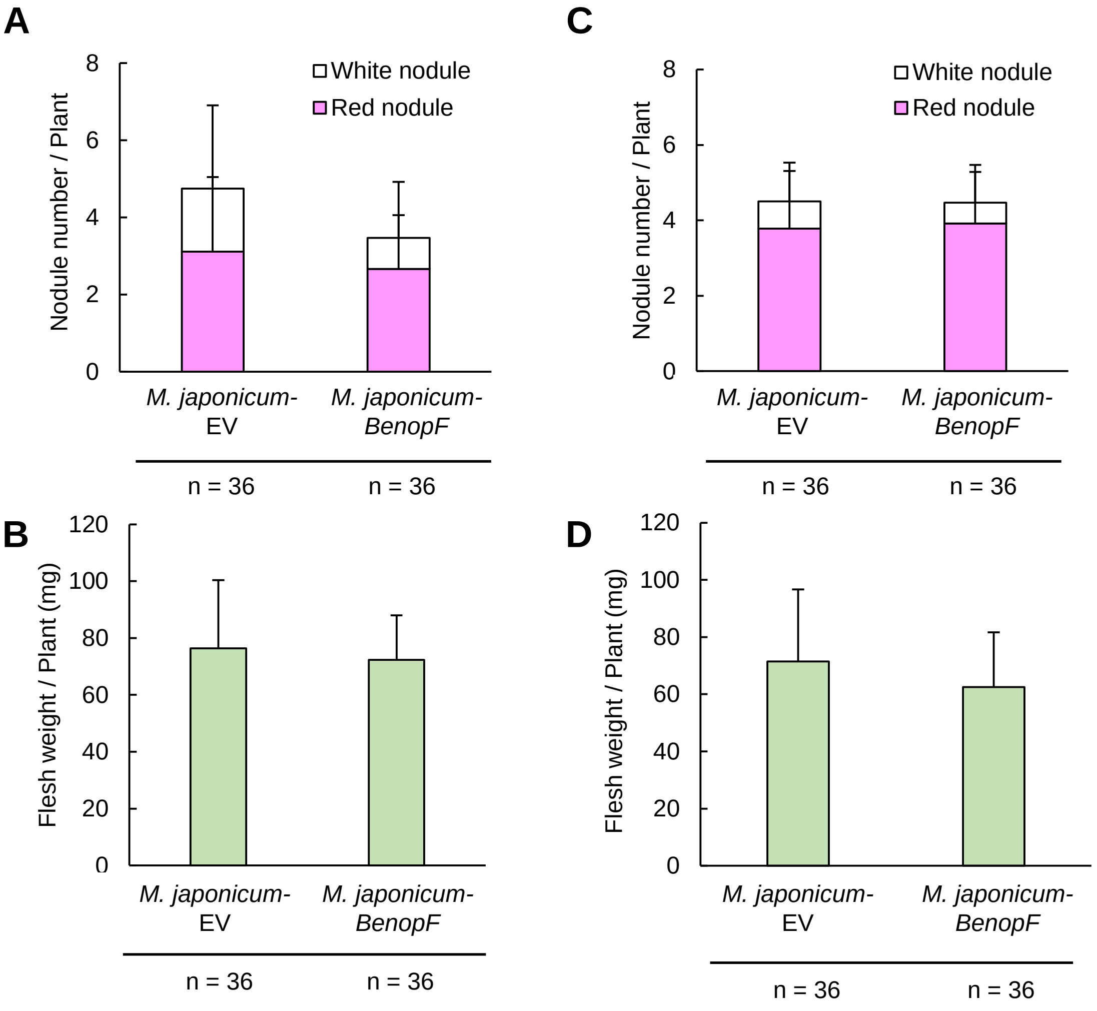

Fig. S16 Symbiotic phenotypes of *L. burttii* and *L. japonicus* MG-20 inoculated with *M. japonicum* MAFF303099 carrying NopF of *B. elkanii* USDA61. (A, B) Nodule number and (C, D) fresh weight of (A, C) *L. burttii* and (B, D) *L. japonicus* MG-20 inoculated with *M. japonicum* carrying the GFP-expressing plasmid pHC60 (*M. japonicum*-EV) or pHC60-*BenopF* (*M. japonicum*-*BenopF*) at 21 days post inoculation. All tests were performed three times with 12 plants each time.

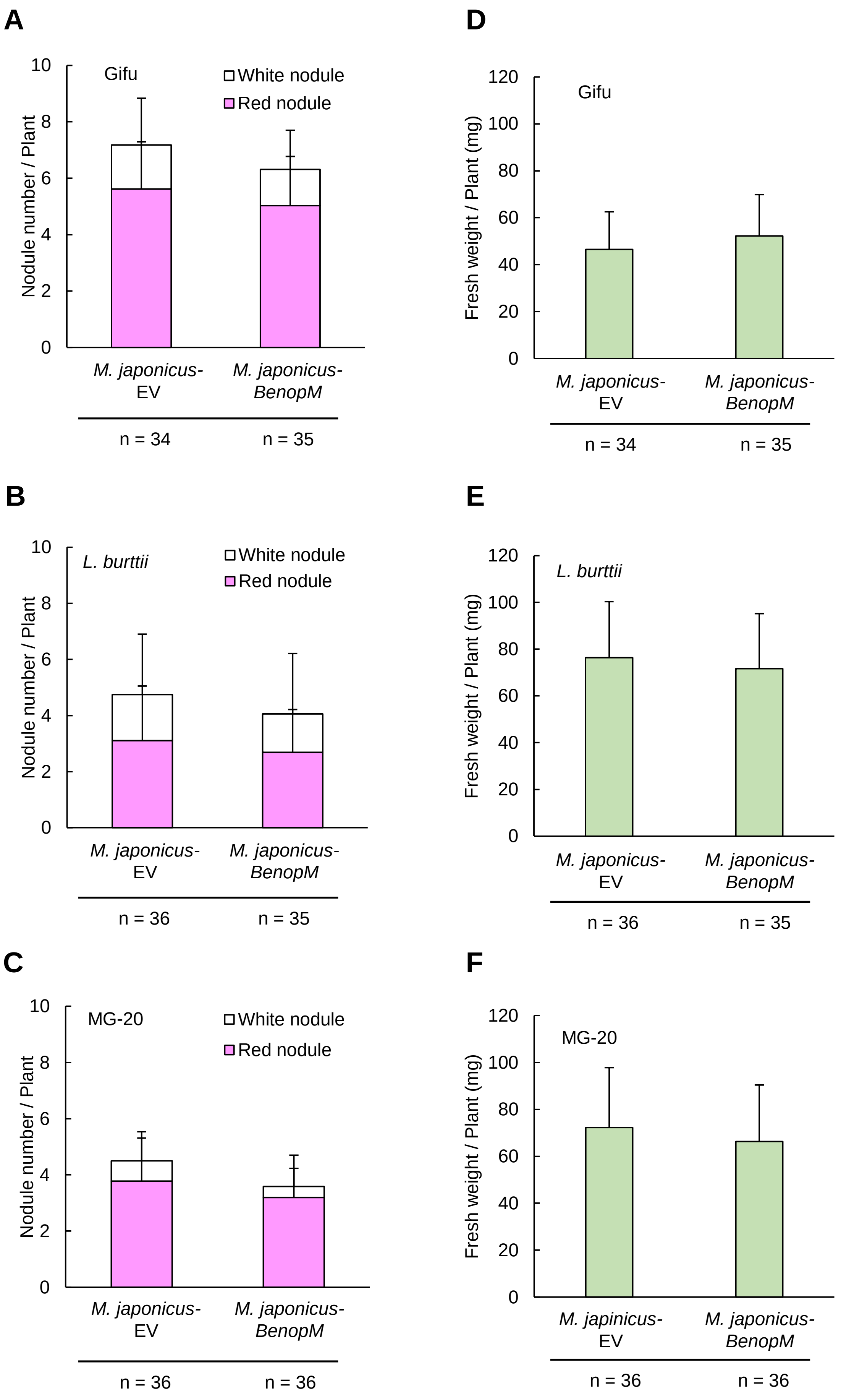

Fig. S17 Kusakabe et al.

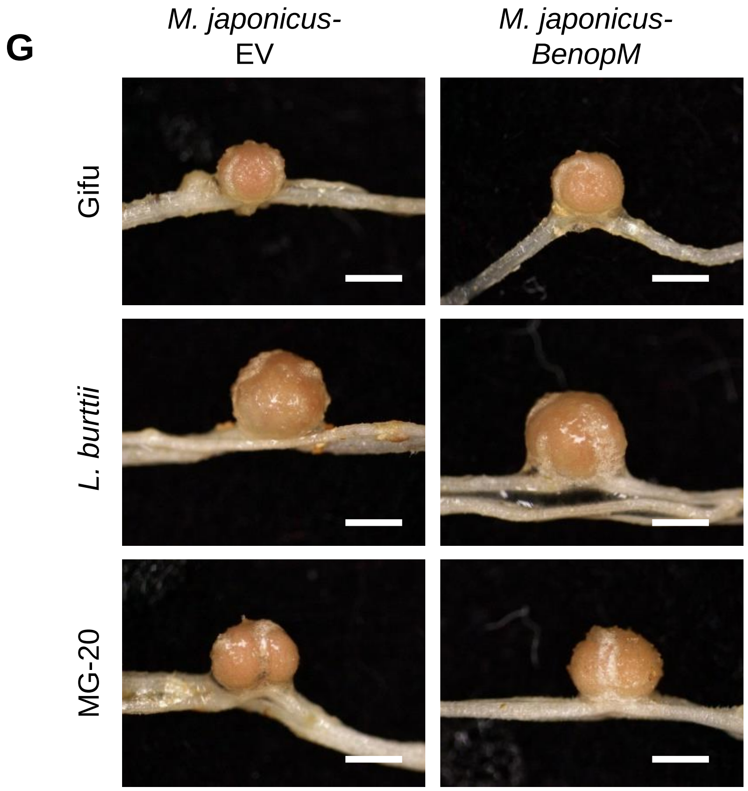

Fig. S17 Symbiotic phenotypes of *Lotus* accessions inoculated with *M. japonicum* MAFF303099 carrying NopM of *B. elkanii* USDA61. (A–C) Nodule number and (D–F) fresh weight of (A, D) *L. japonicus* Gifu, (B, E) *L. burttii*, and (C, F) *L. japonicus* MG-20 inoculated with *M. japonicum* carrying the GFP-expressing plasmid pHC60 (*M. japonicum*-EV) or pHC60-*BenopM* (*M. japonicum-BenopM*) at 21 days post inoculation. All tests were performed three times with 10 to 12 plants each time. (G) Root nodules of *Lotus* accessions inoculated with *M. japonicum*-EV or *M. japonicum-BenopM* at 21 days post inoculation. Scale bars = 1 mm.

Table S1. Matched peptides of candidate effector proteins

Accession	Description	Matched peptides
BE61_80730	Nodulation outer protein NopP	AGDGDSATQAPR<br>EEYSDFVSK<br>FFSYK<br>FLGSDIQSIASSR<br>LAEMGFVHVGR<br>LGDETVGLLR<br>NDLTSVVDLR<br>NENDEWQR<br>QFDSSDEDDSTEYYYLER<br>SSFNDGLWR<br>VGGPVR<br>VTHPLVENAGDILLEHQLR<br>YSLESNPPISEIDR
BE61_78180	Unknown protein	AGQPGGNASQQGDFER<br>ALGLHPAEDAGQPVAEGAR<br>APSFAGPSGGAQELR<br>FLPPYLVR<br>FQLDAPAQVPQSSLR<br>LREVGAGNTLSADLR<br>SAPAPEFR<br>SSSPDQAPTGAGR<br>YQQILEANR<br>YSATFGPGGR<br>YSLPDAEPGVQAR
BE61_80150	Nodulation outer protein NopX	AAIEALQQDPELFYAIGSQGDGR<br>AGDLSGFSDHHPQVAAFQEQR<br>DTTNDHSASQVTASTESTK<br>FFDFGGGHTVDSGNISR<br>NDFNQFFNNMSGANR<br>NLSLADFR<br>NQDAFFGNGDLTR<br>QLTEELTQEAR<br>SILPPEIR<br>SLSPEVR
BE61_80320	Nodulation outer protein NopM	EQFFQLASGASER<br>FAQEPGAQDYAR<br>GPVMHQVLER<br>IAPEDHVAMER<br>LGGTVNYGNEAFR<br>LNADVEDGVYDGR<br>LVDAMGDEFQAR<br>NPLQLLHIAPDMR<br>NQEAAEFPDYLATR<br>WHPWESVLR

Table S1 continued

BE61_77110	Nodulation outer protein NopP	EAAEDYGSTR<br>ELFPGR<br>FVSTR<br>IAENPR<br>IFQNDHEIMR<br>TEGGYSMNEFHDDR<br>TVALLR<br>WTTNSAGEWQR<br>WYPLNEESK<br>YYSYNLGNK
BE61_76200	Unknown protein	ASLALLGR<br>GASSWPSGLTAEGHDQDSPR<br>HQQTPNLGAVVR<br>LTEALELGAGGPHPEDAVTR<br>MDPFNSINPFDR<br>QLSEPGTSMAR<br>WGLLPDETQPVKEYDLR<br>YTALFVPAAAMR
BE61_80180	Nodulation outer protein NopA	ADKVGASHSSTAGGAAGTATAAAGSAAAEAAGEAAFNR<br>DVELR<br>QLAALTASSTK<br>VADERVQ<br>VGASHSSTAGGAAGTATAAAGSAAAEAAGEAAFNR<br>VVSTELSTVK<br>VVSTELSTVKK
BE61_91540	Unknown protein (NopF)	DALAGTSGADGAAFTAALAAGPASSAFDKR<br>ILSIENLGR<br>TNYNNWPSDGQSVDQQR<br>VSSNIGNAQSVSLFTDANER
BE61_78310	Unknown protein	GYFGDIK<br>IGGATWNVFEGFNGHK<br>SALEWIK
BE61_51850	Unknown protein	AQAIPENER<br>EVLFLGGNLDDFR<br>YEQAPWNIWGGQR
BE61_80070	Nodulation outer protein NopL	AYSPYLDAR<br>MDFNSISPTNTSPQPDSPSAPAGPAGFEHQLR<br>TTSFTMLGMPHTAEFR
